# An amphipathic peptide with antibiotic activity against multidrug-resistant Gram-negative bacteria

**DOI:** 10.1038/s41467-020-16950-x

**Published:** 2020-06-23

**Authors:** Alysha G. Elliott, Johnny X. Huang, Søren Neve, Johannes Zuegg, Ingrid A. Edwards, Amy K. Cain, Christine J. Boinett, Lars Barquist, Carina Vingsbo Lundberg, Jason Steen, Mark S. Butler, Mehdi Mobli, Kaela M. Porter, Mark A. T. Blaskovich, Sergio Lociuro, Magnus Strandh, Matthew A. Cooper

**Affiliations:** 1 Centre for Superbug Solutions, Institute for Molecular Bioscience, The University of Queensland, Queensland, QLD 4072 Australia; 2 School of Life Science and Technology, Weifang Medical University, Weifang, 261053 China; 3 Orphazyme, Ole Maaloesvej 3, 2200 Copenhagen, Denmark; 4 Wellcome Sanger Institute, Hinxton, UK; 5 Department of Molecular Sciences, Macquarie University, NSW 2109 Australia; 6 Helmholtz Institute for RNA-based Infection Research (HIRI), Würzburg, Germany; 7 Faculty of Medicine, University of Würzburg, Würzburg, Germany; 8 Statens Serum Institut, Copenhagen, Denmark; 9 School of Chemistry and Molecular Biosciences, The University of Queensland, Queensland, Qld Australia; 10 Centre for Advanced Imaging, The University of Queensland, Queensland, Qld Australia; 11 Adenium Biotech ApS, Ole Maaloesvej 3, 2200 Copenhagen, Denmark; 12 BioVersys AG, Hochbergerstrasse 60C, Technology Park, 4057 Basel, Switzerland; 13 Trinity College Dublin, Dublin, Ireland

**Keywords:** Drug discovery, Antibiotics

## Abstract

Peptide antibiotics are an abundant and synthetically tractable source of molecular diversity, but they are often cationic and can be cytotoxic, nephrotoxic and/or ototoxic, which has limited their clinical development. Here we report structure-guided optimization of an amphipathic peptide, arenicin-3, originally isolated from the marine lugworm *Arenicola marina*. The peptide induces bacterial membrane permeability and ATP release, with serial passaging resulting in a mutation in *mlaC*, a phospholipid transport gene. Structure-based design led to AA139, an antibiotic with broad-spectrum in vitro activity against multidrug-resistant and extensively drug-resistant bacteria, including ESBL, carbapenem- and colistin-resistant clinical isolates. The antibiotic induces a 3–4 log reduction in bacterial burden in mouse models of peritonitis, pneumonia and urinary tract infection. Cytotoxicity and haemolysis of the progenitor peptide is ameliorated with AA139, and the ‘no observable adverse effect level’ (NOAEL) dose in mice is ~10-fold greater than the dose generally required for efficacy in the infection models.

## Abstract (web summary)

Peptide antibiotics often display a very narrow therapeutic index. Here, the authors present an optimized peptide antibiotic with broad-spectrum in vitro activities, in vivo efficacy in multiple disease models against multidrug-resistant Gram-negative infections, and reduced toxicity.

## Introduction

The prevalence of multidrug-resistant (MDR) Gram-negative bacteria is of grave concern^1,2^. Gram-negative bacteria possess efflux pumps, porins, and antibiotic-impermeable membranes, which together reduce drug concentrations in the cytoplasm, where most tractable targets are present. They have now evolved extensive resistance elements that cover all classical pathways targeted by current antibiotics: cell wall, folic acid, protein, RNA, and DNA synthesis. Although directly targeting and disrupting the bacterial membrane can potentially circumvent many of these resistance mechanisms, today only one class of such membrane-targeting antibiotics has been approved for treatment of drug-resistant Gram-negative infections: the structurally related lipopeptides colistin (polymyxin E) and polymyxin B. Unfortunately, these have a very narrow therapeutic index, with nephrotoxicity and other adverse off-target effects seen in humans even at the minimum doses needed to achieve efficacy^3,4^. Resistance to these ‘last resort’ antibiotics is increasingly common, with one mechanism recently found to be disseminated by a plasmid-mediated *mcr-1* gene now found in multiple bacterial species over multiple geographies^5,6^. Antimicrobial peptides that also exert immunomodulatory effects are, in theory, an alternative to traditional antibiotics. For example, the defensins cathelicidin LL-37 and its murine ortholog mCRAMP weakly inhibit bacterial growth and viability in vitro, but modulate and coordinate innate immune responses with a much greater impact on bacterial burden and survival seen in vivo^7,8^. These and other peptides that affect immunomodulation to clear bacterial infections are extensively discussed in a recent review of alternatives to traditional antibiotics^9^.

‘Rules’ that define the physicochemical properties of small molecules that favor penetration and retention within the cytoplasm of Gram-negative bacteria were well summarized over a decade ago^10^. Cell penetrant Gram-negative antibiotics tend to be small (< 600 Da) and polar (low cLogD_7.4_)^10^. More recently, this analysis was extended to include compound globularity, positive charge, and structural rigidity^11^. We questioned whether similar guidelines could be developed for peptide antibiotics, for which there is a wealth of published in vitro activity, but little translation to pre-clinical models, toxicology, and clinical development^12–15^.

The antibiotic arenicin-3 was used as an exemplar to enhance for Gram-negative activity and in vivo efficacy with concomitant minimization of toxicity. Arenicins are small antimicrobial peptides (AMPs) isolated from the coelomocytes of marine polychaeta lugworm *Arenicola marina*^16^. Arenicin-3 is a more recently identified member of the arenicin family that contains two disulfide bonds forming a 21-residue amphipathic β-hairpin^17^. It exhibits potent and rapid antimicrobial activity in vitro against various MDR and extensively drug-resistant (XDR) pathogenic Gram-negative bacteria (minimum inhibitory concentration [MIC] of 1 μg mL^−1^ against *Escherichia coli*), but it is also cytotoxic and induces hemolysis of human erythrocytes. A close analog, NZ17074, has been reported to induce cell death through membrane interruption in *Candida albicans*^18^, and cause disruption of cell wall synthesis, and DNA/RNA damage, in both *E. coli* and *Salmonella enterica* serovar Enteritidis^19,20^.

We show herein that structure-based design can be applied to antimicrobial peptides to minimize basicity while retaining membrane translocation properties, successfully generating a broad-spectrum antibiotic with efficacy in murine models of Gram-negative bacterial infection, accompanied by ameliorated toxicity and hemolysis compared to the progenitor.

## Results

### Structure-guided optimization of antibiotic specificity

**Fig. 1:**
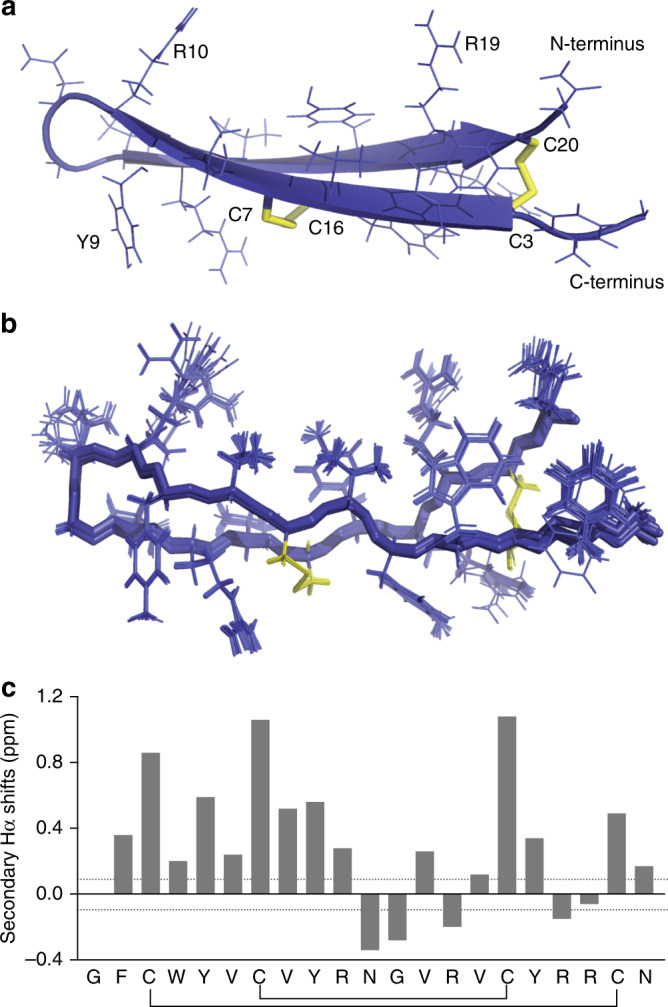
3-Dimensional NMR solution structure of arenicin-3. **a** Structure displayed in cartoon form showing the classic β-hairpin assembly created by anti-parallel β-sheets and two stabilizing disulfide bonds (yellow) across the sheet, key residues and the N and C terminus labeled for orientation. **b** Overlay of 20-lowest energy structures, aligned by backbone. **c** Secondary Hα shifts (variation from random coil). Structure deposited as PDB 5V0Y and BMRB 30259 and images created using PyMol. Structural refinement statistics are displayed in Table 1.

We first determined the NMR solution structure of arenicin-3 (Fig. 1a, b; PDB5v0y; BMRB30259, Table 1). The peptide structure is stabilized by two rigid disulfide bridges Cys3-Cys20 and Cys7-Cys16, which link the two β-strands (Cys3-Asn10 and Arg13-Cys20) forming an anti-parallel β-sheet, connected by a type I′ β-turn (Asn11/Gly12). The pattern of nuclear Overhauser effect (nOE) interactions, variation from random coil values of the secondary chemical shift of carbon and hydrogen atoms (Fig. 1c), and the chemical shift indices (Supplementary Table 1) suggested the peptide adopted a slightly twisted β-hairpin configuration, with 4 hydrogen bonds evenly spaced (every second residue) further stabilizing the β-sheet pinned by the disulfide bonds (Fig. 1, Supplementary Table 2). The distortion created by the right-handed twist of the two-stranded β-sheet allowed the molecule to adopt an amphipathic conformation^15^, where the hydrophobic side of the β-sheet was shielded from contact with a polar solvent, similar to other β-hairpin AMP structures such as tachyplesin-1 (PDB1wo0) and polyphemusin-1 (PDB 1rkk^21^).

**Table 1:** NMR and refinement statistics for arenicin-3 solution structure.

	Arenicin-3^a^
NMR distance and dihedral constraints^b^	
Distance constraints	
Total NOE	381
Intra-residue	102
Inter-residue	279
Sequential (|*i* – *j* | = 1)	125
Medium-range (|*i* – *j* | < 5)	46
Long-range (|*i* – *j* | ≥ 5)	108
Total dihedral angle restraints	38
*ϕ*	19
*ψ*	19
Disulfide bond restraints	12
Total number of restraints per residues	20.5
Structure statistics	
Violations (mean and s.d.)	
Distance constraints (Å)	0 ± 0
Dihedral angle constraints (°)	0 ± 0
Average pairwise r.m.s. deviation^c^ (Å)	
Heavy	0.61
Backbone	0.11
Stereochemical quality^d^	
Residues in most favored Ramachandran region (%)	100.0 ± 0.0
Ramachandran outliers (%)	0 ± 0
Unfavorable sidechain rotamers (%)	1.3 ± 2.4
Clashscore, all atoms^e^	3.06 ± 0.6
Overall MolProbity score	1.23 ± 0.24 (98th percentile)

^a^Arenicin-3 NMR solution structure has been deposited as PDB5V0Y and BMRB30259.

^b^Structurally relevant constraints as defined by CYANA.

^c^Pairwise r.m.s. deviation was calculated among 20 refined structures.

^d^Stereochemical quality as reported by Molprobity 4.2 (http://molprobity.biochem.duke.edu).

^e^Clashscore is defined as number of steric overlaps >0.4 Å per thousand atoms. All statistics are given as mean ± S.D.

With this structural data in hand, we then designed, synthesized, and characterized a series of arenicin-3 analogs and evaluated their activity, toxicity, and membrane selectivity. Firstly, we produced an alanine scan series by replacing individual residues independently with alanine. Secondly, we removed and/or mutated 1–4 residues at varying positions (Supplementary Data 1). In each case, no cysteine residues were mutated, in order to maintain the integrity of the peptide backbone and the secondary structure that is required for antimicrobial activity^20^. Finally, as peptides that interface with membranes can possess different tertiary and even secondary structures at the membrane interface compared to those in free solution, we also prepared an isotopically labeled peptide and solved the high-resolution structure on lipid bilayer membrane nanodiscs using NMR spectroscopy^22^. The membrane structure binding pose posited a lipophilic and hydrophilic face orientation with the long axis almost parallel to the membrane bilayer plane, and with C and N termini making stronger interactions with the membrane compared to the turn residues. This result helped to refine our amphiphilicity model, which suggested that changes in the β-turn region and the C and N termini proximal to the second disulfide bridge were the most likely to affect activity and cytotoxicity of the peptide.

### Broad-spectrum antibiotic activity

The activity of the progenitor arenicin-3 and newly designed derivatives was initially assessed by broth microdilution (BMD) assays determining MICs against a panel of reference strains of Gram-negative and Gram-positive bacteria, and yeasts (Supplementary Data 2). A subset of active peptides showed 2- to 16-fold increased potency compared to arenicin-3 against the *E. coli* reference strain ATCC 25922 and the New Delhi Metallo-β-lactamase-1 (NDM-1) producing strain of *Klebsiella pneumoniae* BAA-2146 (Table 2). Those derivatives were further tested against additional MDR and XDR Gram-negative bacteria, Gram-positive bacteria, and yeasts (Supplementary Data 2 provides MICs, Supplementary Data 3 provides strain information). Although broad-spectrum potency was limited against both Gram-positive bacteria (AA139 MIC against *Staphylococcus aureus* of 32–64 μg mL^−1^ and MIC of 2 μg mL^−1^ against *Bacillus subtilis* and *Listeria monocytogenes*), and yeasts (AA139 MIC of 64 μg mL^−1^ against * C. albicans* and MIC of 1–4 μg mL^−1^ against *Cryptococcus neoformans*), AA139 possessed widespread Gram-negative activity with potency against MDR and XDR isolates, including those resistant to the last-resort antibiotics (MIC 2 μg mL^−1^ against a polymyxin-resistant (PmxR) *Pseudomonas aeruginosa* clinical isolate, 0.5 μg mL^−1^ against an XDR/PmxR *Acinetobacter baumannii* clinical isolate, 0.5–0.125 μg mL^−1^, against two *mcr-1-*positive *E. coli* clinical isolates). AA139 was more active than polymyxin B and colistin against a single PmxR *K. pneumoniae* isolate from Greece, but did display elevated MICs compared to its potent activity seen against the other PmxR species (i.e. colistin MIC of >64 μg mL^−1^, polymyxin B MIC of 32 μg mL^−1^, and AA139 MIC of 16 μg mL^−1^). The elevation in AA139 activity observed with this strain, but not other PmxR isolates, may be due to different mechanisms of resistance leading to PmxR across the tested species. AA139 and selected antibiotic comparators were then profiled against two large panels of clinical isolates (a US panel of 331 isolates from 2010 to 2012 and a worldwide panel of 445 isolates from 2011 to 2013) containing a wide selection of non-MDR, MDR, and XDR clinical isolates with multiple drug resistance mechanisms, such as ESBL (extended spectrum β-lactamase producing), IPM (imipenem)-resistant, and Carb (carbapenem)-resistant, Supplementary Table 3. In these population inhibitory studies MIC_90_ values for AA139 were 1.0 μg mL^−1^ (*E. coli*), 4.0 μg mL^−1^ (*K. pneumoniae*), 8.0 μg mL^−1^ (*P. aeruginosa*), and 2.0 μg mL^−1^ (*A. baumannii*) (Table 3). The results show that AA139 in vitro antimicrobial activity is independent of resistance phenotype (Table 3). MICs were also determined in agarose plates against the *E. coli* reference strain ATCC 25922, which were consistent with the BMD MICs. Among the peptides tested, AA139 (V8A, Y9R, V13A) showed broad potent activity against all tested Gram-negative strains, including MDR and XDR strains. The in vitro antibacterial activity of the arenicin-3 variants was further tested in the presence of human serum and lung surfactant against *E. coli* ATCC 25922 (Supplementary Table 4) and against a panel of clinical isolates (Supplementary Table 5), to evaluate the potential free-drug concentration expected in in vivo models. Across the series of arenicin-3 analogs an 8- to 16-fold loss of activity was seen in serum-induced reversal against *E. coli* (human serum 50% v/v), yet only 2- to 8-fold loss of activity was seen in the presence of lung surfactant (5% SURVANTA® v/v), with the exception of NZ17125 that displayed 64-fold reduced activity in both serum and lung surfactant. Mild cytotoxicity (CC_50_ ~110–300 μg mL^−1^) against HepG2 (hepatocellular carcinoma liver), HEK-293 (embryonic kidney), and HK-2 (kidney proximal tubule) human cell lines was observed for only 3 out of 10 compounds analyzed (Table 2). Of these, only NZ17125 (Y5R, V8A, Y9S, V13L) was found to show toxic effects against all three cell lines. The same peptide, along with arenicin-3, and NZ17126, also displayed mild hemolysis, with HC_10_ values (concentration at which 10% hemolysis is induced) in the range of 181–232 μg mL^−1^ (Table 2). Notably, AA139 was neither cytotoxic nor hemolytic at concentrations 250- to 300-fold higher than the MIC against NDM-1 *K. pneumoniae* and 2000- to 2400-fold higher than the MIC against *E. coli* ATCC 25922. To further evaluate the in vitro toxicity of AA139, it was tested against human primary hepatocytes (Source Data). The assay showed AA139 to have an IC_50_ of 130 μM (330 μg mL^−1^) following 24 h incubation, confirming the specificity of Gram-negative antibacterial activity over general cell toxicity, with 300-fold and 2400-fold higher IC_50_ over antibacterial activity against *K. pneumoniae* and *E. coli*, respectively.

**Table 2:** Arenicin-3 and its analogs assessed for in vitro antimicrobial activity and toxicity.

Peptide	Amino acid sequence	*E. coli* ATCC 25922	*K. pneumoniae* BAA-2146 (NDM-1)	HEK-293	HepG2	HK-2	HRBC
		MIC	MIC	CC_50_	CC_50_	CC_50_	HC_10_
Arenicin-3	GFCWYVCVYRNGVRVCYRRCN	1	2	271	>300	170	204
AA139	GFCWYVC**AR**RNG**A**RVCYRRCN	0.125	1	>300	>300	>250	>300
NZ17125	GFCW**R**VC**AS**RNG**L**RVCYRRCN	0.125	2	250	299	110	181
NZ17126	GFCWYAC**AK**RNG**L**RVCYRRCN	0.125	1	>300	>300	>250	232
NZ17143	GFCW**N**VCV**R**RNGVRVC**H**RRCN	0.25	4	>300	>300	>250	>300
NZ17160	GFCWYVCV**K**RNGVR**S**CYRRCN	0.125	2	>300	>300	>250	>300
NZ17211	GFCW**N**VCVYRNGVR**I**C**H**RRCN	0.5	8	>300	>300	>250	>300
NZ17224	GFCW**H**VC**AR**RNGVRVCYRRCN	0.06	0.5	>300	>300	>250	>300
NZ17228	GFCW**R**ACVYRNGVR**A**CYRRCN	0.125	0.5	>300	>300	>250	>300
NZ17230	GFCW**R**ACVYRNGVRVCYRRCN	0.25	1	223	>300	>250	>300

Antimicrobial activity displayed as MIC μg mL^−1^, mammalian cell cytotoxicity displayed as CC_50_ μg mL^−1^, and red blood cell hemolysis as HC_10_ μg mL^−1^ (concentration inducing 10% hemolysis). MIC values are the mode of ≥6 independent biological replicates, and toxicity values are the mean of *n* = 2. Amino acid changes vs. arenicin-3 are bold underlined. Source data are provided as a source data file.

**Table 3:** AA139 assessed for antimicrobial activity against two clinical isolate panels.

US isolates 2010–2012								
Species	Description	*n*	AA139^a^	GEN	CFZ	MER	CIP	COL
*E. coli*	Non-MDR	24	2	8	2	0.03	**>4**	**4**^b^
	MDR	31	0.5	**>32**	**>32**	**16**	**>4**	0.5^b^
*K. pneumoniae*	Non-MDR	24	4	8	1	0.06	**>4**	**16**^b^
	XDR	55	2	**>32**	**>32**	**>16**	**>4**	0.5^b^
*P. aeruginosa*	Non-MDR	41	8	**>32**	**32**	**16**	**>4**	4
	XDR	37	4	**>32**	**>32**	**>16**	**>4**	2
*A. baumannii*	Non-MDR	69	2	**>32**	**>32**	**>16**	**>4**	2
	MDR	19	1	**>32**	**>32**	**>16**	**>4**	1
	XDR	31	0.5	**>32**	**>32**	**>16**	**>4**	**32**

Worldwide isolates 2011–2013								
Bacteria	Resistance	*n*	AA139^a^	GEN	CFZ	MER	LEV	POL
*E. coli*	^c^	112	0.5	**32**	**32**	0.03	**16**	0.03^b^
*K. pneumoniae*	^c^	114	4	**>32**	**>32**	**>16**	**>32**	0.06^b^
*P. aeruginosa*	^c^	111	8	**>32**	**>32**	**>16**	**>32**	0.25
*A. baumannii*	^c^	108	2	**>32**	**>32**	**>16**	**>32**	0.12

Antimicrobial activity of AA139 and comparator antibiotics against Gram-negative bacterial panels is displayed as MIC_90_ μg mL^−1^. Top: 331 isolates selected from US isolates 2010–2012 panel; bottom: 445 isolates selected from worldwide isolates 2011–2013 panel. Source data are provided as a source data file.

MIC in bold indicates resistance (CLSI breakpoints).

*S* susceptible, *MDR* resistant to 3–4 classes, *XDR* resistant to >4 classes, *CFZ* ceftazidime, *CIP* ciprofloxacin, *COL* colistin, *GEN* gentamycin, *LEV* levofloxacin, *MER* meropenem, *POL* polymyxin B.

^a^No breakpoints defined.

^b^No CLSI breakpoint defined, EUCAST COL breakpoint applied.

^c^MIC_90_ calculated for Worldwide isolates per species including non-MDR, MDR, and XDR strains of each species (Supplementary Table 3).

### Membrane binding and permeation

**Fig. 2:**
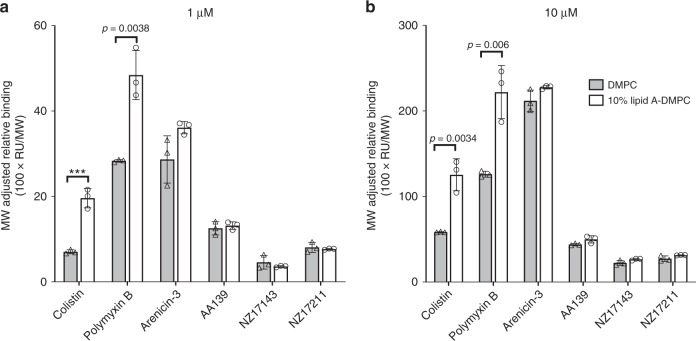
Binding of arenicin-3 variants to lipids as monitored by SPR. Comparison of the propensity of arenicin-3, AA139, NZ17143, NZ17211, and other membrane binding antimicrobials, colistin and polymyxin B, to bind to various model membrane lipid systems. DMPC (gray) is compared to DMPC with 10% *E. coli* lipid A (white) at 1 µM **a** and 10 µM **b**. Mean data of *n* = 3 per test group is plotted with error bars shown as SEM. Significant difference in binding affinities can be seen for colistin and polymyxin B between the two lipid systems; however, not for arenicin-3 variants. Statistical comparison performed with Prism 8 using Student’s *t*-test, all differences between means with *p* < 0.01 are indicated; ***p ≤ 0.001. RU response unit, MW molecular weight. Source data are provided as a source data file.

A previous report by Yang et al.^20^ showed specific binding of NZ17074 to lipopolysaccharide (LPS), and molecular modeling suggested specific binding to the monophosphate group of lipid A via hydrogen bond formation. However, our data show that, unlike other membrane permeable antibiotics (such as polymyxins), the binding of arenicin-3 to the membrane was not dependent on lipid A. Surface plasmon resonance (SPR) experiments were used to compare arenicin-3 and analogs for their binding affinities to 1,2-dimyristoyl-*sn*-glycero-3-phosphocholine (DMPC) lipids alone and DMPC combined with *E. coli* lipid A at a molar ratio of 9:1 (Fig. 2, Supplementary Fig. 2). There was no significant difference between the propensity for arenicin-3 variants to bind to DMPC with lipid A over DMPC alone, in stark contrast to colistin and polymyxin B.

Permeabilization activity against membranes of *E. coli* was investigated using NPN (1-*n*-phenylnapthylamine) and DiSC3(5) uptake assays, which report bacterial outer membrane (OM) permeabilization^23^ and cytoplasmic membrane depolarization^24^, respectively. The fluorescence intensity of NPN markedly increased after adding arenicin-3 variants, like the positive control membrane permeating peptides melittin and polymyxin B (Supplementary Fig. 3a). In the DiSC3(5) assay, all arenicin-3 variants showed relatively weak ability to depolarize the cytoplasmic bacterial membrane (Supplementary Fig. 3b), as compared to melittin and polymyxin B, which were again used as positive controls. Finally, to determine whether the arenicin-3 variants permeabilize the cytoplasmic membrane, they were tested in combination with the SYTOX^TM^ Green fluorescent probe. *E. coli* cells incubated with arenicin-3 variants and SYTOX^TM^ Green were monitored by flow cytometry, showing cell permeabilization and ultimately cell death after 60 min of incubation (Supplementary Fig. 3c).

We further investigated bacterial membrane disruption using transmission electron microscopy (TEM). Clear membrane disruption and cytoplasmic leakage was seen in *P. aeruginosa* after treatment with arenicin-3 variants, an effect also seen, albeit to a lesser extent, in *E. coli* (Supplementary Fig. 4). Mid-log phase *E. coli* and *P. aeruginosa* cultures were incubated with 32 µg mL^−1^ (>16-fold MIC) of arenicin-3 or NZ17143 for 40 min. Most *E. coli* cells were intact after arenicin-3 treatment; however, membrane debris was observed surrounding the cells (Supplementary Fig. 4b, c). Furthermore, mild membrane disruption and cytoplasmic leakage was observed following treatment of *E. coli* with NZ17143 (Supplementary Fig. 4d, e). More significant membrane disruption and cytoplasmic leakage was observed in *P. aeruginosa* (Supplementary Fig. 4g–j). These results suggest that membrane disruption may have a role in the antibacterial mode of action for arenicin-3 variants.

### Arenicin-3 exposure affects ATP release from cells

ATP-efflux as measured by a luminescent microbial cell viability assay indicated rapid leakage of ATP after addition of arenicin-3 and AA139 to mid-phase cultures of *E. coli* K12 MG1655 as compared to piperacillin and colistin sulfate (Supplementary Fig. 5). Piperacillin and colistin induced approximately a third less ATP release in the first 10 min compared to arenicin-3 and AA139. By 30 min, arenicin-3 and AA139 displayed 4-fold more ATP release than piperacillin, and 2-fold more than colistin. At 60 min of incubation at the top three concentrations of 8-fold, 16-fold and 32-fold MIC, arenicin-3 showed ~93% and AA139 showed ~90% ATP release, colistin showed ~80% release of ATP, and piperacillin showed ~20% release of ATP. In addition, at 60 min arenicin-3 and AA139 only reached their maximum ATP release at 4-fold and 8-fold MIC, respectively, whereas at the same time point colistin reached its maximum ATP release at its MIC.

### Low spontaneous and induced resistance in vitro

The propensity for AA139 treatment to induce spontaneous resistant mutants was evaluated by in vitro agar dilution. The **s**pontaneous mutation frequencies in two strains each of *K. pneumoniae* (#3010 & ST258), *P. aeruginosa* (UNT138-1 & ATCC 27853), and *E. coli* (AID#172 & ATCC 25922) were determined under selection of 4× and 8× MIC of AA139 compared to the antibiotic colistin. The frequency of spontaneous mutants was ≤1.6 × 10^−^^9^ for AA139 against the six strains at 4-fold MIC, and ≤6.7 × 10^−10^ at 8-fold MIC. Subsequent testing of putative mutants showed that the MIC values for all selected organisms were within one 2-fold dilution of the parent strain (unexposed to AA139). In contrast, the colistin-treated groups resulted in a frequency of spontaneous mutants up to 75-fold higher in *K. pneumoniae* and *E. coli* (from 1.2 × 10^−^^7^ to 3.9 × 10^−7^ at 4-fold MIC), though rates of resistance in *P. aeruginosa* were similar (Supplementary Table 6).

**Fig. 3:**
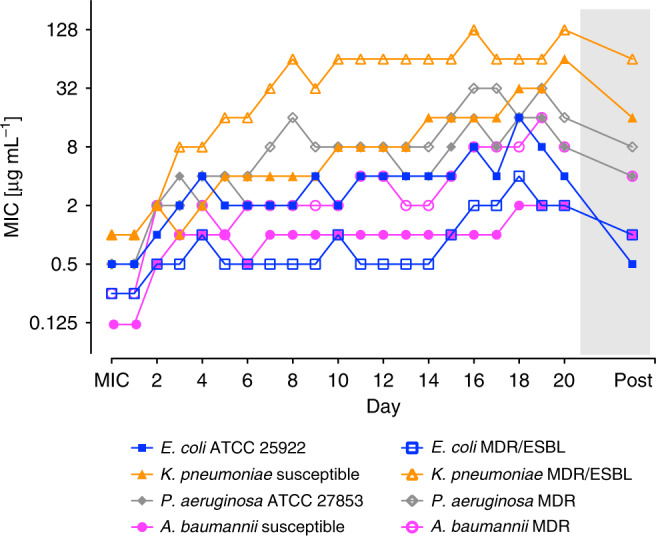
**Serial passage resistance induction of AA139 against susceptible and MDR Gram-negative bacteria**. MIC values for AA139 before and after each of 20 passages (1 passage per 24 h, *n* = 1) treated with 0.5× MIC μg mL^−1^ (of previous passage). Bacteria that grew at the highest concentration of AA139 on the final passage were passaged a further 3 times on drug-free agar plates before determining the final MIC by BMD (“Post”, gray shading). Tabulated MIC values for AA139 and arenicin-3 are presented in Supplementary Table 7. Strains used in this study are detailed by (^#^) in Supplementary Data 3.

Arenicin-3 and AA139 were then challenged over a 20 day serial passage BMD assay to determine the rate of potential resistance induction (Fig. 3, Supplementary Table 7). Two isolates, one drug susceptible and one MDR, of each of four species, *E. coli*, *K. pneumoniae*, *A. baumannii*, and *P. aeruginosa* were treated over 20 serial passages in broth with either arenicin-3 or AA139. Following the 20 days of passages, a further 3 drug-free passages were conducted to assess stability of any induced resistance. Both arenicin-3 and AA139 showed the least induction of resistance against *E. coli* compared to the other species, with a non-stable increase in MIC of 16-fold and 8-fold for arenicin-3 and AA139 against *E. coli* ATCC 25922 on day 20, respectively. For *E. coli*, MDR/ESBL both peptides induced an 8-fold increase in MIC over 20 days, which reverted to 4-fold following the drug-free passage resulting in a final MIC of 1 μg mL^−1^. The most affected organism was *K. pneumoniae* with a stable increase in resistance following 20 passages plus 3 drug-free passages of 16- to 64-fold in the presence of the two peptides.

Finally, AA139 was challenged against a highly diverse Gram-negative panel of over 330 isolates with varying susceptibility and resistance mechanism profiles (Supplementary Table 3) in BMD in vitro assays. No cross-resistance was identified between AA139 and the last-resort antibiotic colistin (Supplementary Fig. 6). In particular, against the colistin-resistant isolates the MIC of AA139 did not surpass 0.5 μg mL^−1^ for *E. coli* with a colistin MIC of 4 to 16 μg mL^−1^, 2 μg mL^−1^ for *A. baumannii* with a colistin MIC of 8–≥32 μg mL^−1^, 8 μg mL^−1^ for *P. aeruginosa* with a colistin MIC of >32 μg mL^−1^, and 4 μg mL^−1^ for all but two *K. pneumoniae* isolates with a colistin MIC of 8–≥32 μg mL^−1^.

### Targeting phospholipid transportation

**Fig. 4:**
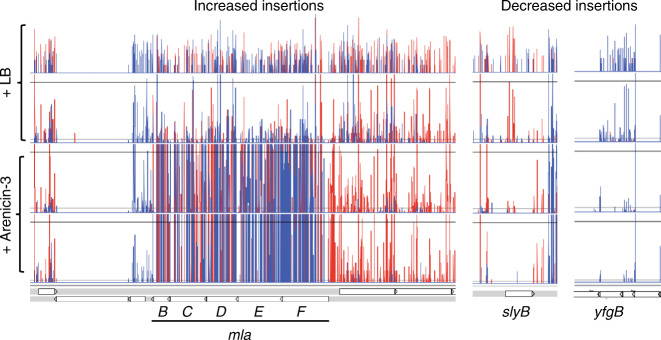
**TraDIS reads representing transposon insertions across the*mla*operon (B-F)**, ***slyB*and*yfgB*in UPEC**. The arenicin-3-treated library is represented by the bottom two rows and the controls with LB only, the top two rows. Insertion positions of Tn*5* mutations at each bp are represented by vertical lines, the depth represented by the height of the line and the direction of insertion, the color (forward and reverse are red and blue, respectively) across the entire *mla* operon. Note, *mlaA* is not shown here as it is located separately but also displayed the same pattern of insertions.

To investigate the genetics underlying adaptation of bacteria to arenicin exposure, we performed transposon directed insertion sequencing (TraDIS) analysis on a uropathogenic *E. coli* (UPEC) strain. A random Tn*5* mutant library of ST131 UPEC strain NCTC 13441, containing ~380,000 unique insertions^25^, was grown in duplicate overnight with, and without, a sub-inhibitory concentration of arenicin-3 (0.25× MIC). TraDIS analysis of the relative mutant abundance of the UPEC treated with arenicin-3, compared to the untreated control, revealed that the *mlaA-F* operon had the most significantly increased mutant population compared to any other genes (increased insertions of log_2_fold change of 4.95-5.3), indicating increased *mla* mutant fitness in the presence of arenicin-3 (Fig. 4, Supplementary Data 4). The *mla* operon regulates the expression of phospholipid transport system *mlaABCDEF*, which stabilizes and restores the integrity of the membrane^26,27^. This suggests that arenicin-3 may cause pressure on the *mla* operon and therefore dysregulates the UPEC phospholipid transport pathways. We speculate that this pressure is either evidence of the bacteria attempting to overcome the lipid asymmetry caused by arenicin-3 disruption or possibly that the *mla* genes are a direct target of arenicin-3 in UPEC, and by removing *mla*-encoded gene products, cell fitness in the presence of arenicin-3 could increase. Genes involved in adaptation to arenicin-3 selection (with decreased insertions in the treated sample) from the TraDIS analysis included outer membrane proteins *slyB* and *yfgB* (Fig. 4), which are involved in maintaining membrane integrity and symmetry (supporting the posited primary mode of action on the membrane), as well as more general first line antibiotic resistance genes, such as efflux pump genes *macAB* and *tolC* that displayed decreased insertions. In support of the TraDIS data suggesting the *mla* operon is a target during arenicin-3 treatment, we performed whole-genome sequencing of the previously mentioned 20 day serial passage of the *E. coli* ATCC 25922 isolate in the presence of arenicin-3, and found a single point mutation in the *mlaC* gene (T33G; L11R, Supplementary Fig. 7).

### In vivo profile of AA139

**Fig. 5:**
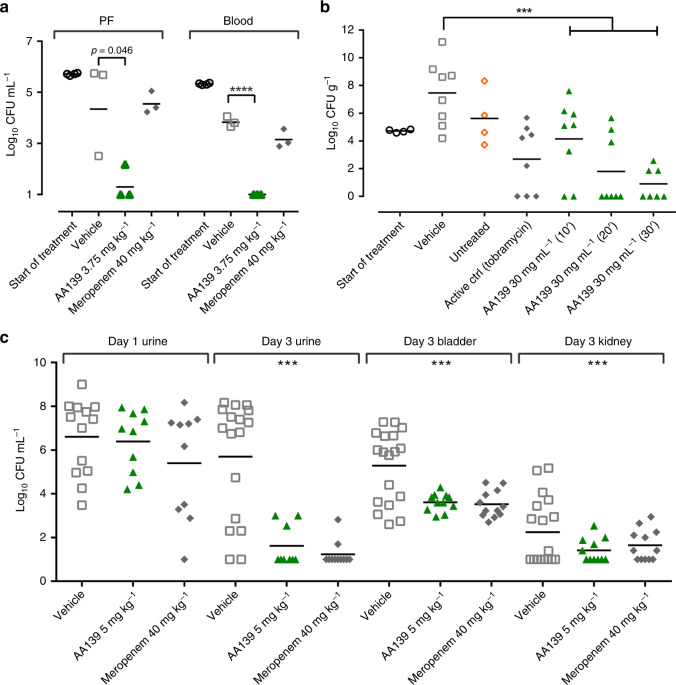
In vivo **peritonitis, pneumonia and UTI murine models for AA139**. **a** Peritonitis model dosed at 3.75 mg kg^−1^ against MDR *E. coli* AID#172 (inoculated in the lateral lower quadrant of the abdomen by single I.V dose) with CFU mL^−1^ measured in the peritoneal fluid (PF) and the blood after 5 h post infection (*n* = 4 for start of treatment groups and *n* = 3 for other groups). **b** *P. aeruginosa* pneumonia model treated with aerosolized AA139 for 10, 20, or 30 min duration in an aerosol exposure chamber at 2, 12, and 24 h post infection. The bacterial load (CFU g^−1^ lung tissue) was measured at 34 h post infection (*n* = 4 for start of treatment group, *n* = 7 for AA139 30 min treatment group and *n* = 8 for other groups). **c** Bacterial load (CFU mL^−1^) in the urine of UTI murine model infected with ESBL producing *E. coli* DSA 443 before treatment (day 1), and bacterial load (CFU mL^−1^) in the urine, bladder, and kidney after last treatment (day 3). Animals were treated twice-daily with AA139 at 5 mg kg^−1^ or meropenem at 40 mg kg^−1^ i.v. (*n* = 12). Horizontal bar indicates geometric mean burden of each treatment. Statistical comparison performed with Prism 8 using one-way ANOVA, Dunnett’s multiple comparisons test, and all differences between means with *p* ≤ 0.05 are indicated: ***p ≤ 0.001; ****p ≤ 0.0001. Source data are provided as a source data file.

The in vitro activity spectrum of action, low cytotoxicity, and retention of some activity in the presence of serum and surfactant suggested that the optimized peptide AA139 could be a lead candidate suitable for testing in models of MDR Gram-negative infections. We therefore evaluated the in vivo efficacy of AA139 against *E. coli* AID#172 MDR clinical isolate (AA139 MIC = 0.25 μg mL^−1^, resistant to ampicillin, ceftazidime, aztreonam, gentamicin, and ciprofloxacin), using a single intravenous (i.v.) dose (0.06–7.5 mg kg^−1^) in a neutropenic murine peritonitis model (Supplementary Fig. 8a, b). Neutropenic mice were inoculated intraperitoneally with ~10^6^ CFU of *E. coli*, with compound administered 1 h post infection, and colony counts in peritoneal fluid and blood determined after killing 5 h later. The ED_50_ was 1.85 mg kg^−1^ in peritoneal fluid and 1.55 mg kg^−1^ in blood at 5 h post treatment. Significant reduction in bacterial load (colony-forming units, CFU) was seen with a single dose at 3.75 mg kg^−1^, leading to a ~3-log reduction in peritoneal fluid and ~4-log reduction in blood, when compared to the vehicle treatment group (Fig. 5a). In the same model, meropenem treatment (40 mg kg^−1^, meropenem MIC = 0.125 μg mL^−1^) resulted in a slight reduction of the CFU levels both in peritoneal fluid and blood compared to vehicle treatment, but this reduction was not statistically significant. The ED_50_ was also determined for the same isolate in a neutropenic murine thigh infection model (Supplementary Fig. 8c), with two intravenous doses of AA139 administered 6 h apart (0.6–15 mg kg^−1^), with 5 mg kg^−1^ polymyxin B as a positive control. Neutropenic mice were inoculated in the thigh with 0.05 mL of ~1–5 × 10^7^ CFU mL^−1^ of *E. coli*, with compound administered 1 and 7 h post infection, and colony counts in thighs determined after killing 24 h post first treatment. The ED_50_ was 3-fold higher 4.8 mg kg^−1^. AA139 at 10 mg kg^−1^ (3.61 log reduction compared to saline control) was equivalent to polymyxin B at 5 mg kg^−1^ (3.13 log reduction) and a 5 mg kg^−1^ dose of AA139 was not statistically different (1.94 log reduction), despite a 4-fold higher MIC (AA139 = 0.125 μg mL^−1^, polymyxin B = 0.03 μg mL^−1^).

The efficacy of aerosolized AA139 was tested against *P. aeruginosa* (ATCC 27853) in a pneumonia model. Infected mice were treated with aerosolized AA139 (MIC = 1.0 μg mL^−1^) or tobramycin (MIC = 0.5–2.0 μg mL^−1^) in an aerosol exposure chamber for different durations at 2, 12, and 24 h post infection. Exposure to nebulized AA139 at 30 mg mL^−1^ for 30 min resulted in a substantial reduction of *P. aeruginosa* burden in the lung (6.6-log CFU per gram) when compared with vehicle-treated mice. In addition, in this group 6 of the 7 animals cleared the infection to below the limit of detection (<374 CFU per gram, see methods) of the assay (Fig. 5b). Mice were treated with 7.5 and 15 mg mL^−1^ of AA139 for 10-min exposure, and 30 mg mL^−1^ of AA139 for 10, 20, and 30 min. A statistically significant reduction in *P. aeruginosa* burden was observed in the lungs of all groups treated except the lowest dose, and an increased clearance of bacterial burden was observed as the exposure time was lengthened in the mice exposed to 30 mg mL^−1^ of AA139 (Fig. 5b). A limitation of the experimental setup was that the exact delivered dose could not be determined; however, the results show that AA139 remains active and effective after nebulization and inhalation.

AA139 was also tested against an ESBL producing *E. coli* DSA 443 strain (AA139 MIC = 0.5 μg mL^−1^), in a urinary tract infection (UTI) model. Following infection of the bladder, mice were dosed twice-daily i.v. for 2 days with AA139 at 5 mg kg^−1^ or meropenem at 40 mg kg^−1^, assessing the bacterial burden on day 3 in the urine, bladder and kidneys (Fig. 5c). A significant decrease in infection was observed in the AA139-treated group, with reduction in CFU equivalent to that caused by an 8-fold higher dosage of meropenem (MIC = 0.032 μg mL^−1^). Arenicin-3 and analogs NZ17143 (MIC = 0.5 μg mL^−1^) and NZ17211 (MIC = 2.0 μg mL^−1^) were also tested for efficacy in the UTI model and showed similar promising efficacy at 0.8–12.5 mg kg^−1^. NZ17211 was the most efficacious of the three tested compounds at the lowest dose of 0.8 mg kg^−1^, showing the greatest bacterial burden reductions in urine and bladder (Supplementary Fig. 9).

**Fig. 6:**
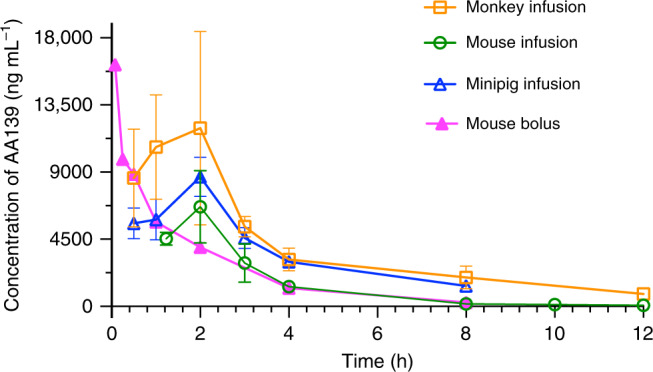
The pharmacokinetic profile across species of systemically administered AA139. Plasma or serum AA139 concentration in mouse, monkey and minipig over time after the initiation of a 10 mg kg^−1^ dose (i.v. continuous infusion over 2 h, or i.v. single bolus injection). Mean values with error bars shown as SEM (mouse i.v. infusion n = 6, mouse i.v. bolus *n* = 3, monkey *n* = 2, minipig *n* = 2; male and female combined for all species) are plotted using Prism 8. Source data are provided as a source data file.

We then investigated the i.v. pharmacokinetic (PK) profile of AA139 in mice, cynomolgus monkeys, and minipigs. AA139 showed rapid and widespread distribution beyond the central compartment, bi-exponential elimination with a half-life of ~2 to 4 h, and moderate clearance consistent with ~20% the rate of liver blood flow, or similar to the glomerular filtration rate, suggesting primarily renal clearance (Supplementary Table 8). Following a 2 h constant i.v. infusion of 10 mg kg^−1^ to the mouse, cynomolgus monkey or minipig, the extent of plasma AA139 exposure varied between species, yet the shape of the plasma concentration versus time curve remained consistent across species (Fig. 6). In contrast, the PK profile of inhaled AA139 exhibited high concentrations of AA139 in the epithelial lining fluid (ELF) and lung tissue of mice, whereas systemic absorption following inhalation administration appeared to be minimal, resulting in very low concentrations in the plasma (Supplementary Table 9) when compared to ELF or lung tissue. Systemic exposure to inhaled AA139 increased in a greater than dose-proportional manner between target doses 5 and 20 mg kg^−1^ per day on both days 1 and 7. Following 7 days of once-daily aerosol administration to mice, AA139 concentrations in ELF were persistent for up to 24 h post-dose, suggesting the potential for once-daily administration in a clinical setting.

AA139 toxicity was evaluated following i.v. administration in single and repeated dose studies in mice, cynomolgus monkeys and minipigs, as well as inhalation administration in mice. In general, the dose-limiting toxicity of systemically administered AA139 appeared related to excessive pharmacological dose, presenting with clinical signs of lethargy, decreased locomotion, swelling at extremities, tremors, and twitching (Supplementary Data 5). Target organ toxicity at high exposure appeared to be limited to the kidney with tubular nephropathy present in the minipig at 20, 25, or 30 mg kg^−1^ per day of AA139 by 2 h i.v. infusion for 7 days, with a dose-related increase in severity. Other tissues were macroscopically and microscopically unremarkable. Both clinical signs and histopathological changes were generally dose-related. In 7-day or 14-day repeated administration of a 2 h i.v. infusion once or twice daily, the no observable adverse effect level (NOAEL) in mice was 20 mg kg^−1^ per day (ca. 10-fold the dose generally required for efficacy in murine infection models), whereas in monkeys the NOAEL was 15 mg kg^−1^ per day when given i.v. over 2 h QD or BID. When given via aerosol inhalation over 7 days, the NOAEL in mice was 20 mg kg^−1^ per day.

## Discussion

We have shown that improved peptide antibiotic analogs can be developed by following a rational design approach accounting for lipophilicity, charge and amphipathicity^12,28–30^. Detailed structure–activity and structure–toxicity relationship analysis of the progenitor antibiotic arenicin-3 suggested that: (i) lipophilicity is a key contributor to the cytotoxicity of the peptide; (ii) swapping Tyr5, Val6 Val13, and Val15 with more lipophilic residues, and swapping Val8, Tyr9, and Tyr17 with more hydrophilic residues reduced cytotoxicity, while retaining antibacterial activity; (iii) replacing or deleting Arg18 and Arg19 significantly reduced antibacterial activity; (iv) substituting aromatic residues (Phe2, Trp4, Tyr5, Tyr9, and Tyr17), and to a lesser extent aliphatic residues (Val6, Val8, Val13, and Val15) with Ala was beneficial for antibacterial activity, and finally; (v) substituting Tyr17 with His decreased cytotoxicity. This analysis, combined with the membrane models and NMR solution structures, led to peptide AA139 (H-GFCWYVC**AR**RNG**A**RVCYRRCN-OH; changes vs. arenicin-3 in bold; V8A, Y9R, V13A) as a promising lead.

The new variants retained potent activity against a range of MDR and XDR clinical isolates, including those resistant to the last-resort polymyxin antibiotics, and were effective against the high priority pathogens *E. coli, K. pneumoniae, A. baumannii* and *P. aeruginosa* (full list of strains in Supplementary Data 3). In particular, AA139 displayed promising efficacy in several pathogenic Gram-negative infection in vivo models and decreased mammalian toxicity compared to the native arenicin-3. Mode of action investigations showed that arenicin activity was consistent with bacterial membrane binding and disruption of membrane integrity. TraDIS analysis and genetic analysis of forced induction of resistance isolates suggested the *mla* operon, which maintains outer membrane asymmetry via retrograde phospholipid transport, could be associated with arenicin’s mechanism of action in *E. coli*. TraDIS showed that *mlaA-F* mutants were fitter than wild-type *E. coli* during arenicin-3 selection, suggesting that by removing any part of this phospholipid transport system, arenicin-3 cannot act as efficiently. Resistance induction experiments resulted in a SNP in *mlaC*, supporting the hypothesis that arenicin-3 places pressure on this phospholipid transport gene. The *mla* operon has been recently proposed to traffic phospholipids from the inner to outer membrane^31^, which is also consistent with our hypothesis that arenicin-3 directly interacts with phospholipids in the outer membrane as the first step of its mechanism of action and interferes with membrane homeostasis.

Eukaryotic membranes are less negatively charged than prokaryotic membranes due to a more neutral lipid composition and internalization of the electrostatic membrane potential within cytosolic mitochondria. The orthogonal hydrophobic and hydrophilic barrier membranes of Gram-negative bacteria necessitate consideration of subtle differences in phospholipid head group regions in order to effect membrane perturbation and translocation in prokaryotes, but not human cells. We show that a structure-selectivity model based on amphipathicity and overall charge can be used to derive a bacteria-selective peptide antibiotic that is active against recalcitrant MDR and XDR Gram-negative bacteria, with reasonable pharmacokinetic properties and efficacy in vivo. However, derivation of new peptide drugs with appropriate safety margins in animals required for progression to clinical trials is still a challenge.

## Methods

### Peptide synthesis

Arenicin-3 and peptide analogs were manufactured using solid phase peptide synthesis by PolyPeptide Group (Strasbourg, France, and Limhamn, Sweden), on behalf of Adenium Biotech ApS. The physiochemical properties of the peptides were calculated using the ExPASy ProtParam tool^32^.

### NMR solution structure

Arenicin-3 was analyzed at 3.5 mM in 20 mM pH 3.3 phosphate buffer containing 10% D_2_O. All NMR experiments were performed on a Bruker Avance III spectrometer equipped with a cryogenically cooled triple resonance probe operating at a nominal ^1^H frequency of 700 MHz. An excitation sculpting sequence was used to suppress the solvent (H_2_O) resonance. Two-dimensional TOCSY [tm (MLEV17 spin-lock mixing pulses) = 80 ms], NOESY [tm (mixing time) = 300 ms], ^15^N-HSQC, and ^13^C-HSQC were recorded at 25 °C. Chemical shifts were directly or indirectly referenced to the 2,2-dimethylsilapentane-5-sulfonic acid (DSS) signal at 0 ppm. The assignment of proton resonances was carried out using TOCSY and NOESY data using the CCPNMR software^33^. The torsion angles constraints were obtained using Talos program^34^. Structure calculations were performed using CYANA 3.0^35^. Additional upper/lower restraints were applied to form disulfide bonds between Cys3-Cys20 and Cys7-Cys16. All NMR spectra for the hydrogen-deuterium exchange studies were recorded on the spectrometer described above at 25 °C. Spectra were referenced to DSS at 0 ppm. The peptide was re-lyophilized and dissolved in 100% D_2_O and immediately placed in the spectrometer for measurement. 1D ^1^H and 2D TOCSY spectra were recorded at specific time points over a 24 h period. Hydrogen-deuterium exchange rates were measured by integrating each exchangeable amide resonance separately. The temperature dependence of amide proton resonances was derived from 1D and TOCSY spectra recorded on a Bruker ARX 500 MHz spectrometer. Spectra were measured between 15 and 35 °C, in 5 °C increments, and referenced to DSS at 0 ppm. Assignment of the spectra was performed using the program CCPNMR. The NMR solution structure of arenicin-3 is deposited with the Protein Data Bank under accession number PDB5V0Y and with the Biological Magnetic Resonance Data Bank as accession number BMRB30259.

### Determination of antimicrobial activity

Antimicrobial activity of arenicin-3 and its analogs was tested against a panel of bacterial and yeast strains by in vitro broth microdilution (BMD) assay with minimum inhibitory concentration (MIC) determination. Strains were purchased from American Type Culture Collection (ATCC) or obtained from clinical collaborators (full strain profiles provided in Supplementary Data 3). All peptides were prepared in Ringer’s acetate solution, and subsequently diluted fresh in water for each independent assay.

MICs were determined using in vitro BMD assays in 96-well non-binding surface polystyrene plates (NBS; Corning Cat # 3641), with the MIC being recorded as the lowest concentration of peptide in the assay wells that showed no visible bacterial growth. Assays were performed in Mueller Hinton broth (MHB; BD, Cat. No. 211443) for bacteria, and yeast extract-peptone dextrose (YPD; Sigma-Aldrich, Y1500) for yeast. The concentrations of the peptides and comparator antibiotics/antifungals in the assay wells ranged from either 128 or 64 μg mL^−1^, to 0.06 or 0.03 μg mL^−1^ as a 12-point dose response plated as a 2-fold dilution series across the wells of the plate in duplicate for each independent biological replicate, using a final cell density of 5 × 10^5^ CFU mL^−1^ and 2.5 × 10^3^ CFU mL^−1^ for bacteria and yeast respectively. Final assay volumes were 100 μL per well with MICs determined visually following 18-20 h incubation at 37 °C for bacteria, 24 h incubation at 35 °C for *Candida albicans*, and 24 h incubation at 35 °C with resazurin addition (0.006%; Sigma-Aldrich, Cat # R7017) for a further 3 h for *Cryptococcus neoformans*, without shaking. Additional bacterial and yeast susceptibility assays were performed in Cation-Adjusted Mueller Hinton Broth (CAMHB; BD, Cat No. 212322) and Roswell Park Memorial Institute (RPMI) 1640 culture medium (Life Technologies Australia, Cat No. 21870092), respectively, as recommended by CLSI guidelines (M07 11th ed^36^ and M27 4th ed^37^). CAMHB bacterial MICs did not significantly differ to those conducted in MHB, and Yeast MICs in RPMI did not differ to those conducted in YPD (maximum of 4-fold variation, which is acceptable within the error of the assay, data presented in Supplementary Data 2). However, for yeast it was determined that the YPD media was more reliable and robust between repeats and therefore MICs in this media are reported. Human serum and lung surfactant effects were determined using MIC BMD as described above against bacteria with either the addition of 50% (v/v) human serum (human male AB plasma, Sigma-Aldrich Cat # H4522) or 5% (v/v) lung surfactant (SURVANTA®, Abbvie Pty Ltd, Cat # 1039.008) into the growth media^38^. Agarose MICs were determined in MHB with 1% agarose (Bioline molecular grade; Cat no: BIO-41025; high purity, high gel strength). All experiments were performed a minimum of six replicates (*n* ≥ 6).

### In vitro activity profiling and cross-resistance assessment

The in vitro activity of AA139 and comparator antimicrobials was determined in two panels of clinical isolates of *E. coli*, *K. pneumoniae*, *P. aeruginosa,* and *A. baumannii* (Supplementary Table 3), and MIC_90_ presented. The US panel consisted of 331 clinical isolates from 2010 to 2012. It was tested by BMD in accordance with the CLSI guidelines (M07-A9, 2012)^39^. The comparator drugs were ceftazidime, tigecycline, ciprofloxacin, meropenem, colistin (ThermoFisher Scientific), and gentamicin (Sigma-Aldrich). AA139 was diluted in 0.01% acetic acid/0.1% bovine serum albumin. Sterile polypropylene culture cluster round-bottom 96-well plates (Costar # 3879) were used for testing. The worldwide panel consisted of 445 clinical isolates from 2011 to 2013, geographically divided into Asia, Europe, North America, and Rest of World (Middle East, Latin America, Africa, and South Pacific) with each region representing ~25% of the isolates. It was tested by broth microdilution in accordance with the CLSI guidelines (M07-A9, 2012)^39^. The comparator antimicrobials were polymyxin B sulfate, meropenem, gentamicin, ceftazidime (USP), and levofloxacin (Sigma-Aldrich). The test media was CAMHB (Becton Dickinson). AA139 and polymyxin B were tested in media supplemented with 0.002% Tween 80 (Calbiochem). MIC_90_ values were calculated for all or subsets of the isolates. MIC correlation between AA139 and colistin was analyzed with data from the US panel.

### Cytotoxicity and hemolysis assays

HEK-293 human embryonic kidney cells (ATCC CRL-1573), HepG2 liver hepatocellular carcinoma cells (ATCC HB-8065), and HK-2 human kidney proximal tubule cells (ATCC CRL-2190) cell lines were purchased from ATCC. Cytotoxicity of arenicin-3 variants was determined using the Alamar Blue (resazurin; Sigma-Aldrich, Cat # R7017), with 1% FBS for HK-2 and 10% FBS for HEK-293, and HepG2^40^. Cells were seeded as 2000 cells per well in black wall clear bottom 384-well plates (Corning^®^, Costar^®^, Cat # 3712). The cells were incubated for 24 h at 37 °C, 5% CO_2_. Then peptides were added into each cell containing well. After 24 h incubation, 5 μM resazurin were added per well and incubated at 37 °C for 2 h. Then the fluorescence intensity (FI) was read using a Polarstar^®^ Omega plate reader with excitation/emission 560/590 and CC_50_ values were determined using Prism 8 following curve fitting and applying the following equation:

$${\mathrm{Percentage}}\,{\mathrm{viability = (FI}}_{{\mathrm{TEST}}}{\mathrm{ - FI}}_{{\mathrm{Negative}}}{\mathrm{/FI}}_{{\mathrm{UNTREATED}}}{\mathrm{ - FI}}_{{\mathrm{Negative}}}{\mathrm{)}} \times {\mathrm{100}}{\mathrm{.}}$$

Hemolytic activity was measured against isolated human erythrocytes from anonymously donated fresh human blood^15^. Isolated erythrocytes were adjusted to 1 × 10^9^ blood cells mL^−1^ in phosphate-buffered saline (PBS, pH 7.4), added to the wells of NUNC polypropylene round-bottom 96-well plates (Sigma-Aldrich, Cat # P6866) then up to 300 μg mL^−1^ of compound was added per well and incubated for 3 h at 37 °C. Following incubation, samples were centrifuged at 3000 rpm for 20 min, and the supernatants, 100 μL, were carefully collected for measurement. The degree of hemolysis in the samples was determined by measurement of the absorption of the supernatants at 540 nm using the Polarstar-Omega plate reader. 1% Triton-X100 was used as positive control for inducing 100% hemolysis. HC_10_ and HC_50_ values were determined using Prism 8 following curve fitting. The use of human blood (sourced from the Australian Red Cross Blood Service) for hemolysis assays was approved by the University of Queensland Institutional Human Research Ethics Committee, Approval Number 2014000031. All in vitro toxicity assays were performed for two independent biological replicates as an 8-point dose response of 3-fold dilution series in triplicate with the highest test concentration of 300 μg mL^−1^.

### ADME-Tox; in vitro toxicity

Cell viability of human primary hepatocytes against AA139 was assessed by fluorometric readout of Alamar Blue following 24 h incubation at 37 °C of human plateable cryopreserved hepatocytes and AA139 as an 8-point dose response, 0.1–300 μM, as described by Nociari et al.^41^. Cell viability was calculated as a percentage of the control cells and plotted versus concentration using Prism 8 Software. The IC_50_ value was determined by non-linear regression analysis of the concentration–response curve using the Hill equation. Assay was performed by Eurofins Pharma Discovery Services (Panlabs), St Charles, MO, USA, study number 100049990. Source data are provided as a source data file.

### Surface plasmon resonance

SPR experiments were performed on a Biacore T200 using an L1 sensor chip as previously described^42^. Small unilamellar vesicles (SUVs) were prepared in PBS by sonication and extrusion. Lipids were dissolved in ethanol-free chloroform in 25 mL round-bottom flasks. 10% (mol/mol) of *E. coli* lipid A, diphosphoryl (Sigma-Aldrich, Cat # L5399) was added into 1,2-dimyristoyl-*sn*-glycero-3-phosphocholine (DMPC) (Auspep, Cat # 850345P) solution to make lipid A–DMPC mixtures, which were then deposited as a thin film by removal of the solvent (chloroform) under reduced pressure on a rotary evaporator and dried under high vacuum for at least 2 h. PBS was then added into each flask to give a 1 mM (DMPC) suspension, which was sonicated 5 min for five times. The suspension was passed 17 times through a 50 nm polycarbonate filter in an Avestin Lipofast Basic extrusion apparatus to give a translucent solution of vesicles, which should possess a mean diameter of 50 nm. The SUVs were then injected into the flow cells of the L1 sensor chip for 2000 s at a low flow rate of 2 μL min^−1^ to form a bilayer membrane model on the chip surface. Then, a series of peptide solutions (30–0.33 μM in 3-fold dilution), was injected across the flow cells at a flow rate of 30 μL min^−1^, having an injection phase of 180 s, and a dissociation phase of 300 s. The binding responses (RU) were normalized by molecular weight (MW) of each peptide. The equation 100 × RU/MW was used for normalization. The relative affinities were compared by selecting a reporting time point toward the end of the peptide-to-lipid association phase (*t* = 180 s).

### Membrane perturbation assays

For the investigation of outer membrane permeability, 1-*N*-phenylnaphthylamine (NPN) uptake assay was performed^43,44^. NPN is an uncharged, hydrophobic fluorescent probe that has very weak fluorescence in an aqueous environment. However, it shows strong fluorescence in a hydrophobic interior of a membrane. Upon outer membrane disruption, NPN is able to reach the hydrophobic environment of the membrane, emitting bright fluorescence. NPN was added to 2 × 10^6^ *E. coli* ATCC 25922 cells mL^−1^ (final NPN concentration of 20 μM) and incubated for 15 min with varying concentrations (64–0.015 μg mL^−1^) of lipopeptides with the fluorescence emission intensity recorded (*λ*_exc_ = 340 nm, *λ*_em_ = 405 nm) using a Polarstar-Omega (BMG) spectrophotometer to evaluate NPN uptake by the bacterial cells.

The 3,3′-dipropylthiadicarbocyanine iodide (DiSC3(5)) assay^45^ was employed to investigate cytoplasmic membrane depolarization. DiSC3(5) accumulates in cells on hyperpolarized membranes, and its fluorescence is self–quenched. When the membranes lose potential, the dye is released and emits fluorescence. Mid-log phase (OD ~ 0.6) *E. coli* ATCC 25922 cells were harvested by centrifugation and resuspended in 5 mM HEPES, 20 mM glucose, pH 7.4. The uptake and self-quench of DiSC3(5) dye into bacterial cells was monitored for 30 min. Then compounds in a concentration series were added and the fluorescence intensity (*λ*_exc_ = 620 nm, *λ*_em_ = 670 nm) was measured using a Polarstar-Omega (BMG) spectrophotometer.

Inner membrane permeability was measured using SYTOX^TM^ Green (Invitrogen/Life Technologies, S7020) uptake assay^46^. SYTOX^TM^ Green is a DNA binding dye that is fluorescent when bound to nucleic acids and only enters cells with compromised plasma membranes. Firstly, suspensions of 1 × 10^7^ *E. coli* ATCC 25922 cells mL^−1^ were incubated for 1 h at 37 °C with compounds at varying concentrations. Then SYTOX^TM^ Green was added at 2 μM to the compound-treated *E. coli* cells for 5 min at room temperature. Cells were compared for their fluorescence emission profiles by flow cytometry performed with a BD FACS Canto II (BD Biosciences; *λ*_exc_ = 488 nm, *λ*_em_ = 530 nm) with 30 nm bandpass.

### Electron microscopy

Mid-log phase bacteria (*E. coli* ATCC 25922 and *P. aeruginosa* ATCC 27853) grown in LB broth were adjusted to a density of 5 × 10^8^ CFU mL^−1^. Arenicin-3 variants were added to bacterial suspension at a final concentration of 32 μg mL^−1^ and incubated for 40 min at 37 °C under constant shaking. Bacteria were then centrifuged and frozen using a Leica EM PACT2 high pressure freezer. Frozen samples were fixed in 2% glutaraldehyde using a Leica EM AFS2 freeze substitution processor. Subsequently, the samples were embedded in EPON epoxy-resin and polymerized at 60 °C overnight. Ultrathin-sectioning (80–100 nm) was performed with a diamond knife, using a Leica ultra-cut microtome. The slices were placed on a copper grid (400 squares). Pictures were taken with a JEOL 1011 TEM.

### Measure of ATP release

*E. coli* K12 MG1655 (ATCC 700926) cells (5 × 10^5^ CFU mL^−1^ mid-log phase in CAMHB) were exposed to various MIC-folds (MIC and 1/4×, 1/2×, 2×, 4×, 8×, 16×, 32× MIC) of arenicin-3 (MIC 0.25 μg mL^−1^), AA139 (MIC 0.125 μg mL^−1^), piperacillin sodium salt (Sigma-Aldrich, Cat # P8396; MIC 2 μg mL^−1^), and colistin sulfate (Sigma-Aldrich, Cat # C4461; MIC 0.03 μg mL^−1^). ATP was measured using the BacTiter-Glo^TM^ Microbial Cell Viability Assay (Promega) following the manufacturer’s instructions. The assay was performed for three biological replicates at 37 °C, with luminescence recorded using a Tecan Infinite M1000 Pro plate reader. The fold reduction of ATP was calculated as: Fold reduction ATP = 1 − (*L*_treat_ − *L*_media_/*L*_control_ − *L*_media_); where *L*_treat_ is the luminescence of treated cells, *L*_media_ is the luminescence of CAMHB without cells, and *L*_control_ is the luminescence of untreated cells. Resultant graphs show mean (*n* = 7) and std error for each data point, prepared in Prism 8. DMSO was included as a control as piperacillin was solubilized in DMSO, with final assay concentration of 2.5%.

### Spontaneous frequency of resistance

The spontaneous mutation frequencies in two strains each of *K. pneumoniae* (#3010 & ST258), *P. aeruginosa* (UNT138-1 & ATCC 27853) and *E. coli* (AID#172 & ATCC 25922) were determined under selection of 4× and 8× MIC of AA139 and colistin sulfate (Sigma-Aldrich). The test media was cation-adjusted MHB (CAMHB, Becton Dickinson) supplemented with 2% Noble Agar (Becton Dickinson) and additionally for AA139 with 0.1% BSA. The agar dilution MIC values were determined as recommended by Clinical and Laboratory Standards Institute (CLSI)^47^. Bacterial strains were tested against AA139 and colistin at 4× and 8× MIC as determined by agar dilution testing. The inoculum for the spontaneous mutation plates targeted 10^9^ to 10^10^ colony-forming units (CFU) per plate. The viable count of each suspension was determined by plating serial ten-fold dilutions onto MHB/NA in duplicate. In addition, agar plates without drug were inoculated as positive controls. Plates were inspected for growth at both 24 and 48 h. Colony counts were determined manually. Using the counts at 48 h, the spontaneous mutation frequency was calculated using the following equation: (average number of colonies from duplicate selection plates)/(total number of cells inoculated onto each plate). If there were no colonies on the antibiotic selection plates, the spontaneous mutation frequency was reported as less than the frequency observed with one colony to indicate that the spontaneous mutation frequency was less than the limit of detection (one CFU).

### Serial passage resistance induction studies

Assay performed by Eurofins Inc., Virginia USA. Serial passage MICs were performed in microtiter panels prepared by Eurofins according to SOP 1-P-PR-PRO-9002355. From inoculated microtiter panels, an aliquot of the well with the highest concentration permitting growth was taken and back diluted in fresh media to a turbidity of a 0.5 McFarland standard. This suspension was then further diluted and used to inoculate a fresh MIC panel resulting in a final concentration of 1.5 × 10^6^ CFU mL^−1^. Panels were incubated according to CLSI guidelines (M07-A9)^36^, MICs were recorded and the next inoculum was prepared from the well containing the highest concentration of drug that allowed growth in identical fashion as described above. Twenty repeat passages were performed. Subsequently, stability studies were performed by taking bacteria that grew at the highest concentration of drug on the final passage, passing them three (3) times on drug-free agar plates, and then evaluating the broth microdilution MIC by CLSI methods as described above. With each daily panel inoculation, purity blood agar plates were also inoculated and read to check for contamination and appropriate concentration of inoculum. All experiments were performed as outlined in Eurofins SOP 1-P-PR-PRO-9002386. MICs of evaluated agents were within CLSI QC range^48^ for evaluated ATCC organisms as applicable (at baseline).

### Genetic analysis of serial passage isolates

*E. coli* strains (ATCC 25922 parent strain, 20 day serial passage isolate, 20 day +3 day drug-free passage) were incubated in MHB overnight at 37 °C. Genomic DNA was purified from 3 mL of pelleted cells using the Powersoil DNA isolation kit (MO BIO, Carlsbad, CA). Indexed whole-genome sequencing libraries were prepared with the Nextera XT DNA sample preparation kit (Illumina, San Diego, CA). Pooled libraries were submitted to the Queensland Centre for Medical Genomics (University of Queensland, QLD, Australia) for sequencing on an Illumina MiSeq (Illumina, San Diego, CA). Raw data was de novo assembled using SPAdes v3.10.1^49^ and annotated via the Rapid Annotation using Subsystem Technology (RAST)^50^. RAST enabled the extraction of the protein sequence for MlaC (ABC transporter substrate-binding protein) and identified the L11R amino acid change (nucleotide change: T32G) in day 20 and 20 + 3 isolates. This sequence was further queried on the UniProt database^51^, which revealed this modification in the signal peptide domain is unique to these isolates. Sequences were submitted to NCBI as Bioproject PRJNA511334.

### TraDIS libraries, sequencing, and analysis

Bacterial cells were transformed with a Tn*5* transposon flanking a chloramphenicol selectable marker^52^. A random Tn*5* mutant library of ST131 UPEC strain NCTC 13441, containing ~380,000 unique insertions^25^, was grown in duplicate overnight with, and without a sub-inhibitory concentration of arenicin-3 (0.25× MIC). Sequencing was performed on a HiSeq 2500 Illumina platform and ~2 million single-end reads per sample were generated, and deposited in the European Nucleotide Archive (ENA) under study PRJEB3226. These were then mapped and analyzed for differences in mutant abundance using the Bio-Tradis pipeline^53^ with default settings using minimum read count of 20 and 10% of 3′ sequence trimmed, against ENA reference sequence ERS530440.

### In vivo pharmacology models

Peritonitis and UTI murine models were performed at the Department of Microbiology & Infection Control, Statens Serum Institute, Copenhagen, Denmark, under approval by the National Committee of Animal Ethics, Denmark, and adhered to the standards of EU Directive 2010/63/EU. The mouse pneumonia model was performed by Evotec Ltd, United Kingdom, under UK Home Office Licenses and with local ethical committee clearance. All animal studies were contracted by Adenium Biotech ApS. Mice were housed in polysulfone cages with an acclimatization period of 7 days prior to experiments with air temperature maintained at 22 °C with 55% humidity and light–dark cycles of 12 h light.

Peptides were prepared in Ringer’s acetate pH 6.0 (vehicle) at 34 mg mL^−1^. Comparator antibiotics included meropenem (Meronem®, AstraZeneca), polymyxin B sulfate (Sigma) and tobramycin (Novartis).

The *E. coli* peritonitis in vivo model was conducted using an *E. coli* AID#172 strain in female NMRI mice (Harlan) that were rendered neutropenic by injecting 0.5 mL cyclophosphamide solution (Apodan®) intraperitoneally for 4 days (~200 mg kg^−1^) and 1 day (~100 mg kg^−1^) prior to inoculation of infection. At time −1 h, mice were inoculated intraperitoneally with 0.5 mL of 2 × 10^6^ CFU mL^−1^ *E. coli* AID#172 suspension in the lateral lower quadrant of the abdomen. Mice were then treated i.v. with 10 mL kg^−1^ of a single dose of AA139 (BMD MIC 0.125 μg mL^−1^), doses ranging from 0.06 to 7.5 mg kg^−1^, meropenem (BMD MIC 0.125 μg mL^−1^, 40 mg kg^−1^) or vehicle at time 1 h (*n* = 3 mice per group). CFU counts were determined from blood and peritoneal fluid at 0 and 5 h after treatment. The mice were anesthetized with Zoletil® and blood was collected by axillary cut down. The mice were killed by cervical dislocation, a total of 2 mL sterile saline was injected i.p. and the abdomen gently massaged before it was opened, fluid sampled with a pipette, and collected for CFU counts on agar.

The *E. coli* thigh infection in vivo model was conducted using an *E. coli* AID#172 strain in female NMRI mice (Taconic) that were rendered neutropenic as above. At time −1 h, mice were inoculated intramuscularly in the quadriceps femoral muscle close to the femur of the left hind leg with 0.05 mL of a suspension of ~1–5 × 10^7^ CFU mL^−1^ of *E. coli* (fresh overnight colonies from 5% Horse Blood Agar plate suspended in sterile saline to ~10^8^ CFU mL^−1^, then diluted) with compound administered intravenously 1 and 12 h post infection (injection over 30 s with 10 mL kg^−1^ of 0.6–15 mg kg^−1^ AA139 or 5 mg kg^−1^ polymyxin B). Colony counts were determined in thighs at 0 and 24 h post infection. The mice were killed by cervical dislocation, the skin was removed and the left hind leg from the hip joint to the hock was collected, cut into at least three parts and frozen at −80 °C. After thawing, the thighs were homogenized in 5 mL saline using a Dispomix® Drive. Each sample was then 10-fold diluted in saline and 20 μL spots were applied on 5% blood agar plates. All agar plates were incubated 18–24 h at 35 °C in ambient air.

The *E. coli* UTI in vivo model was prepared using an *E. coli* DSA 443 ESBL strain, in neutropenic outbred OF-1 female mice (Charles River, France). Starting 4 days before inoculation mice were given drinking water with 5% glucose. On day 0 mice were anaesthetized and inoculated with 50 μL bacterial suspension (1 × 10^9^ CFU mL^−1^) of *E. coli* DSA 443 ESBL, via a catheter in the urethra into the bladder. On days 1 and 2, mice were treated i.v. with vehicle (*n* = 18) or with twice-daily administrations of 5 mg kg^−1^ AA139 (*n* = 12) or 40 mg kg^−1^ meropenem (*n* = 12). Urine samples were taken on days 1–3 and on day 3 mice were killed and urine, kidneys, and bladder were collected for CFU counts on agar.

*P. aeruginosa* pneumonia in vivo model was prepared as follows: male CD1 mice were immunosuppressed with cyclophosphamide 200 mg kg^−1^ on day −4 and 150 mg kg^−1^ on day −1. On day 0, mice were infected by intranasal administration with 40 μL of 2.5 × 10^6^ CFU mL^−1^ *P. aeruginosa* ATCC 27853 suspension. Mice were then placed in an acrylic aerosol exposure chamber (0.11 m^3^) and treated at 2, 12, and 24 h post infectious challenge with aerosolized AA139 (*n* = 8 per group), tobramycin (*n* = 8) or vehicle (Ringer’s acetate, *n* = 8) using a nebulizer (Hudson RCI Micro-mist) with compressed air at 0.5–1 bar. Three strengths of AA139 formulation (in Ringer’s acetate) were nebulized for different treatment durations: 30 mg mL^−1^ (30, 20, 10 min); 15 mg mL^−1^ (10 min); and 7.5 mg mL^−1^ (10 min). Tobramycin was nebulized at 4.2 mg mL^−1^ for 20 min. Pre-treatment and untreated control groups were also included (*n* = 4 per group). Mouse weights were recorded once-daily following infection to ensure animals remained within ethical limits and to monitor efficacy of treatment. All mice remaining in the study at 34 h post infection were euthanized and the burden of *P. aeruginosa* in the lungs was determined. Briefly, the lungs were removed and weighed and then homogenized in 2 mL ice cold sterile phosphate-buffered saline using a bead-beater. The lung homogenates were diluted appropriately then quantitatively cultured on to *Pseudomonas*-selective agar and incubated at 37 °C for 24 h before the number of colonies was counted. The limit of detection for the CFU assay was <374 CFU g^−1^, based on 0.1 mL plating neat sample and tissue weight of 0.308 g. All resultant data were analyzed and displayed using GraphPad Prism. The data from the culture burdens were analyzed with an appropriate non-parametric statistical model (Kruskal–Wallis) using StatsDirect statistical software v 2.7.8 and compared to vehicle control.

### Pharmacokinetic and toxicokinetic studies

The studies were performed at Fidelta Ltd, Croatia (mice), under review by the institutional ethics committee (CARE-Zg), and Covance Laboratories UK and Muenster Germany (cynomolgus monkey and minipig) in accordance with the requirements of the Animals (Scientific Procedures) Act 1989 and a maintained local ethical review. All studies were contracted by Adenium Biotech ApS. Mice were housed in polysulfone cages with an acclimatization period of 7 days prior to experiments with air temperature maintained at 22 °C with 55% humidity and light–dark cycles of 12 h light. Cynomolgus monkeys of age range 4–7 years were selected, tested for health, acclimatized for 2 weeks, and housed in single-animal climate controlled stainless-steel cages with a minimum of 8 air changes per hour with air temperature at 19–25 °C and relative humidity 40–70%, and light–dark cycles of 12 h light. Minipigs of age range 4–6 months were selected, tested for health, acclimatized for 2 weeks, and housed in single-animal pens with 15–20 air changes per hour with air temperature at 22–26 °C with light–dark cycles of 12 h light.

*Mouse i.v. injection*: A formulation of AA139 in Ringer’s acetate pH 6.0 (1 mg mL^−1^) was administered to male CD1 mice (Charles River, Italy) at 10 mg kg^−1^ as a 400 μL slow injection into the lateral tail vein. Blood samples were collected from the jugular vein at eight timepoints (*n* = 3 mice): 0.08, 0.25, 0.5, 1, 2, 4, 8, and 24 h. AA139 was quantified in plasma using solid phase extraction (SPE) followed by liquid chromatography with tandem mass spectrometric detection (LC–MS/MS). Pharmacokinetic analysis was performed using Phoenix WinNonlin 6.2. (Pharsight Corporation) software using individual animal data, non-compartmental sparse analysis, and the actual dose as determined from formulation analysis by UPLC.

*Mouse, monkey, and minipig i.v. 2-h infusion*: AA139 formulated in Ringer’s acetate was administered intravenously to CD1 mice, cynomolgus monkeys (*Macaca fascicularis*) and Göttingen A/S minipigs (Ellegaard Göttingen, Denmark) by continuous infusion over 2 h (minipig under anesthesia). Individual dose volumes were based on individual body weight. Blood samples were collected at 0.5, 1, 2, 3, 4, 8, 10, and 12 h after the start of the infusion (*n* = 6 mice, male and female combined; *n* = 2 monkeys, one male and one female; *n* = 2 minipigs, one male and one female). Bioanalytical analysis was performed by SPE followed by LC–MS/MS. The method was validated for an AA139 concentration range of 43.4–21,700 ng mL^−1^. Pharmacokinetic parameters were calculated by standard non-compartmental analysis (NCA) using Phoenix WinNonlin version 6.2. The studies were conducted in compliance with Good Laboratory Practice (GLP) regulations.

### Reporting summary

Further information on research design is available in the Nature Research Reporting Summary linked to this article.

## Supplementary information

Supplementary Information

Description of Additional Supplementary Files

Supplementary Data 1

Supplementary Data 2

Supplementary Data 3

Supplementary Data 4

Supplementary Data 5

Reporting Summary

## Data Availability

NMR solution structure of arenicin-3 is deposited with the Protein Data Bank under accession number PDB5V0Y and with the Biological Magnetic Resonance Data Bank as accession number BMRB30259. TraDIS data are deposited in the European Nucleotide Archive (ENA) under study PRJEB3226. Genome sequences of serial passage isolates are deposited to National Center for Biotechnology Information (NCBI) as Bioproject PRJNA511334. The source data underlying Figs. 2, 5a–c and 6, Tables 2 and 3, Supplementary Figs. 3, 5, 6, 8, and 9, and Supplementary Tables 4 and 5 are provided as a Source Data file. Source data are provided with this paper.

## Source data

Source Data
