# Supplementary material for: An amphipathic peptide with antibiotic activity against multidrug-resistant Gram-negative bacteria
Source: Nat Commun. 2020 Jun 23;11:3184. doi: 10.1038/s41467-020-16950-x (PMC7311426; doi:10.1038/s41467-020-16950-x)
Supplement: Supplementary file 1 — Supplementary Information [file 41467_2020_16950_MOESM1_ESM.pdf]

Supplementary Information

An amphipathic peptide with antibiotic activity against multi-drug-resistant Gram-negative bacteria

Elliott et al.

Arenicin-3					G	F	C	W	Y	V	C	V	Y	R	N	G	V	R	V	C	Y	R	R	C	N			
Tachyplesin-1					K	W	C	F	R	V	C	-	Y	R	-	G	I	-	-	C	Y	R	R	C	R	*		
Polyphemusin-1				R	R	W	C	F	R	V	C	-	Y	R	-	G	F	-	-	C	Y	R	K	C	R	*		
Gomesin						pE	C	R	R	L	C	-	Y	K	Q	-	-	R	-	C	V	T	Y	C	R	G	R	*
Protegrin-1		R	G	G	R	L	C	-	Y	-	C	-	R	R	-	-	-	R	F	C	V	-	-	C	V	G	R	*
Thanatin	G	S	K	K	P	V	P	I	I	Y	C	N	R	R	T	G	-	K	-	C	Q	R	M	*				

Supplementary Fig. 1: Comparison of the structural characteristics of β-hairpin antimicrobial peptides. (adapted from *Edwards et al., ACS Infect. Dis. 2, 442-450*, 2016). Amino acid sequence alignment of selected β-hairpin antimicrobial peptides manually aligned using Cys-Cys as the core, where $_p$E indicates pyroglutamic acid; cystines are marked in yellow; cationic residues in magenta and hydrophobic in green. Boxes: turn region. (*): *C*-terminal amidation.

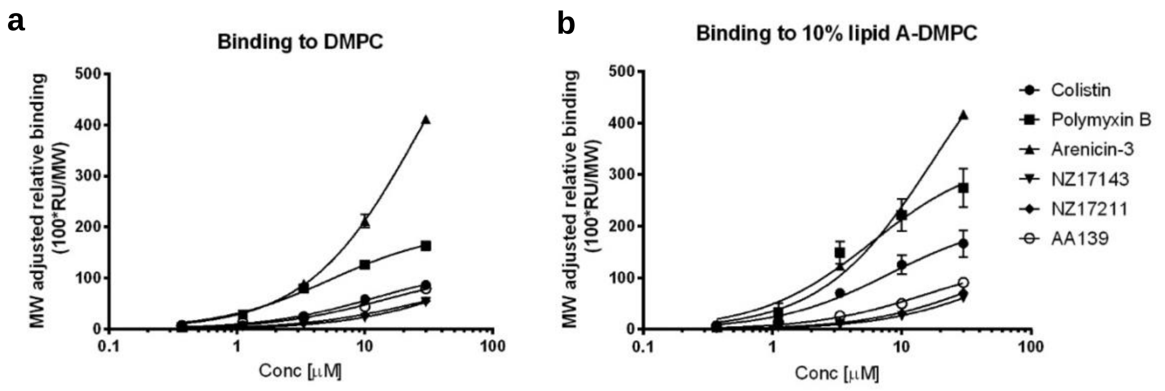

Supplementary Fig. 2: Dose response of peptides binding model lipids monitored by SPR. Relative binding of arenicin-3, arenicin analogues and membrane active antibiotic comparators colistin and polymyxin B to DMPC (**a**) and 10% *E. coli* lipid A/DMPC (**b**) as monitored by SPR. Data analyzed using Prism 8 by nonlinear fit (one site specific binding with hill slope). Each data point is the mean of n=3 (all DMPC values and, NZ17211 and AA139 10% lipid A/DMPC values), or n=6 (colistin, polymyxin B, arenicin-3 and NZ17143 10% lipid A/DMPC values), with error bars displayed as SEM. RU: response unit; MW: molecular weight. Source data are provided as a source data file.

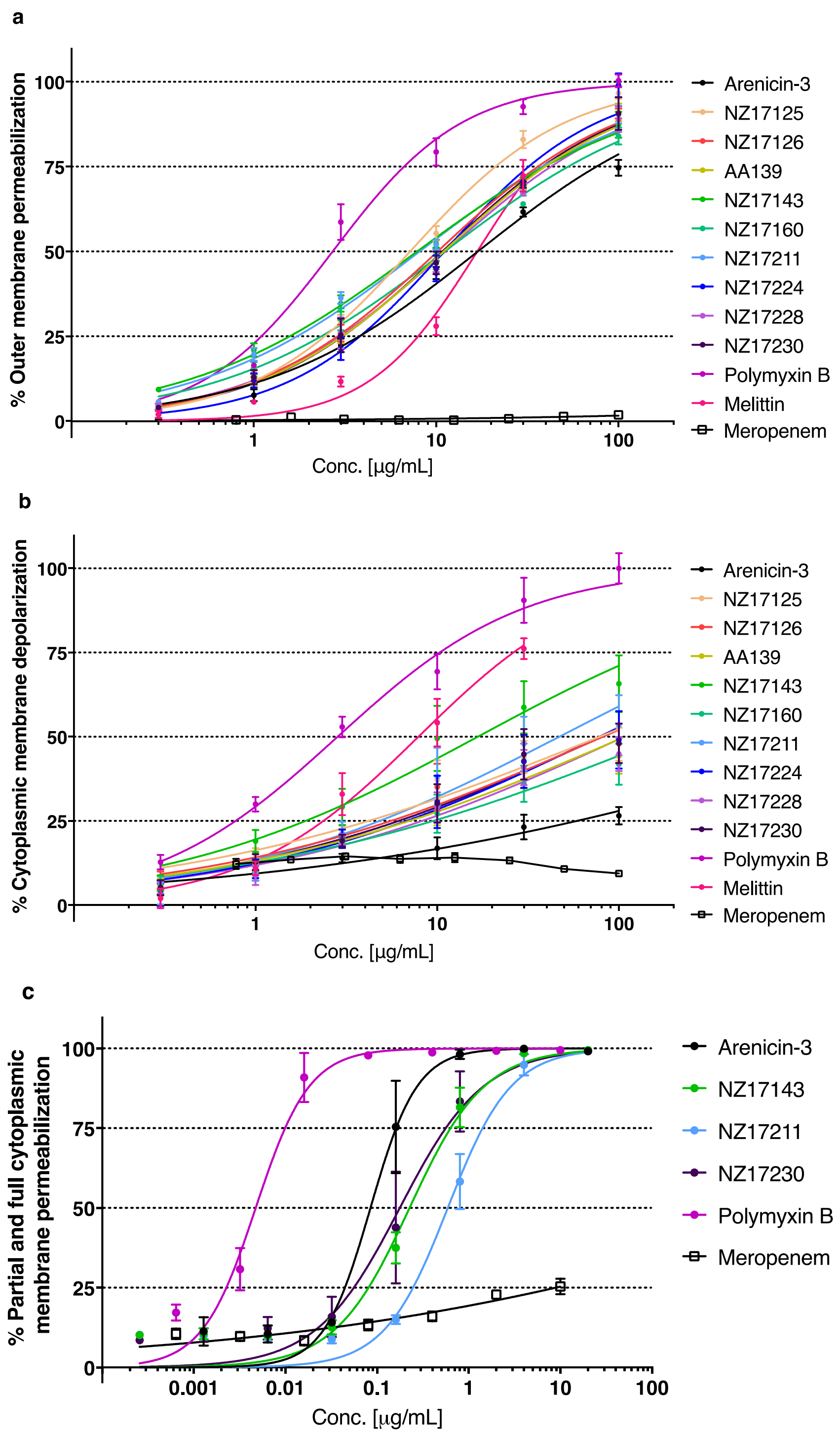

Supplementary Fig. 3: Arenicin peptides show Outer Membrane (OM) permeabilization, cytoplasmic membrane depolarization and permeabilization. (a) NPN uptake assay, **(b)** DiSC3(5) assay, **(c)** SYTOX™ Green assay. Four to ten arenicin-3 analogues assessed with polymyxin B and melittin included as positive membrane permeating peptides and meropenem as negative membrane permeating compound. Data analyzed using Prism 8 by nonlinear fit (one site specific binding with hill slope). Each data point is the mean of n=3 (NPN assay (**a**) all compounds except meropenem, which is n=8), n=4 (DiSC3(5) assay (**b**) all compounds except meropenem, which is n=8), n=3 (SYTOX™ Green assay (**c**) polymyxin B, arenicin-3, NZ17143) and n=4 (SYTOX™ Green assay (**c**) meropenem, NZ17211, NZ17230), with error bars displayed as SEM. Source data are provided as a source data file.

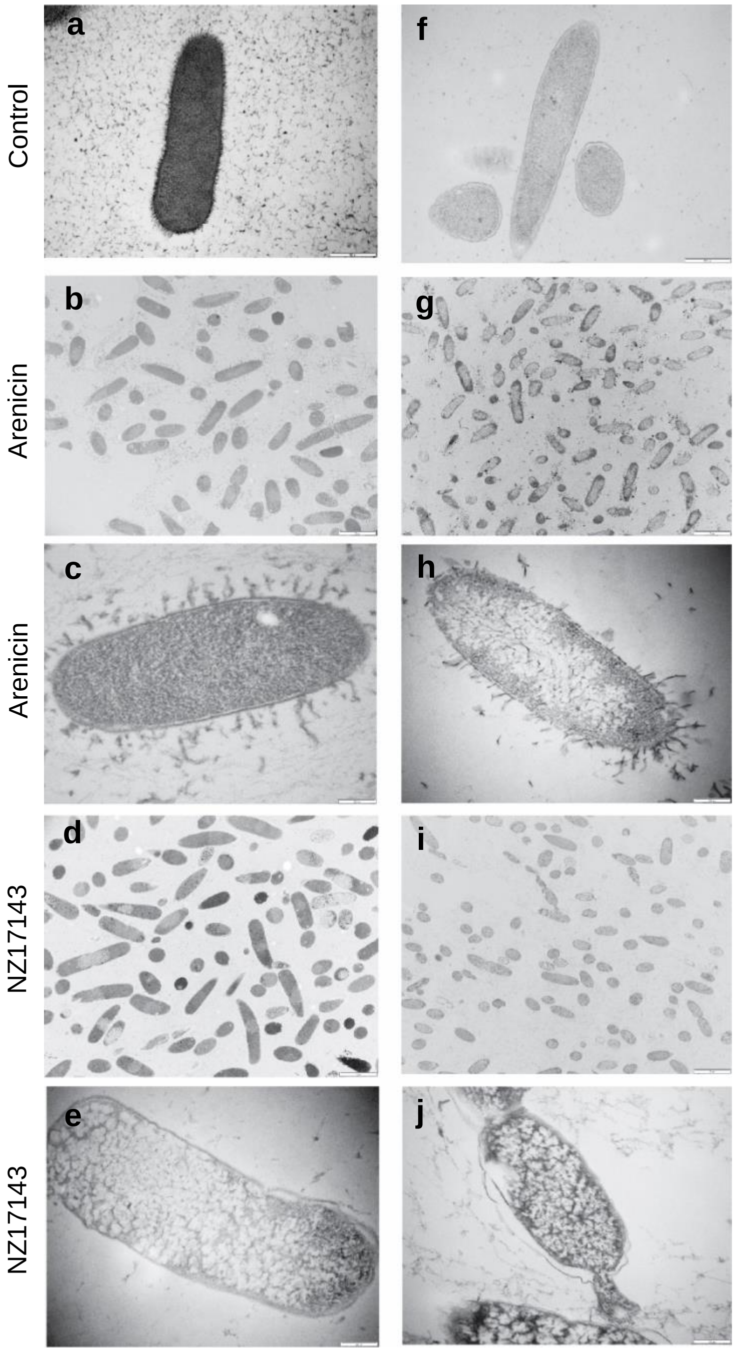

Supplementary Fig. 4: Membrane permeabilization confirmation by TEM studies. *E. coli* ATCC 25922 (left column) treated with arenicin-3 and NZ17143 and *P. aeruginosa* ATCC 27853 (right column) treated with arenicin-3 and NZ17143. Bars: 200 nm (**a, c, e, f, h** and **j**); 2 µm (**b, d, g** and **i**).

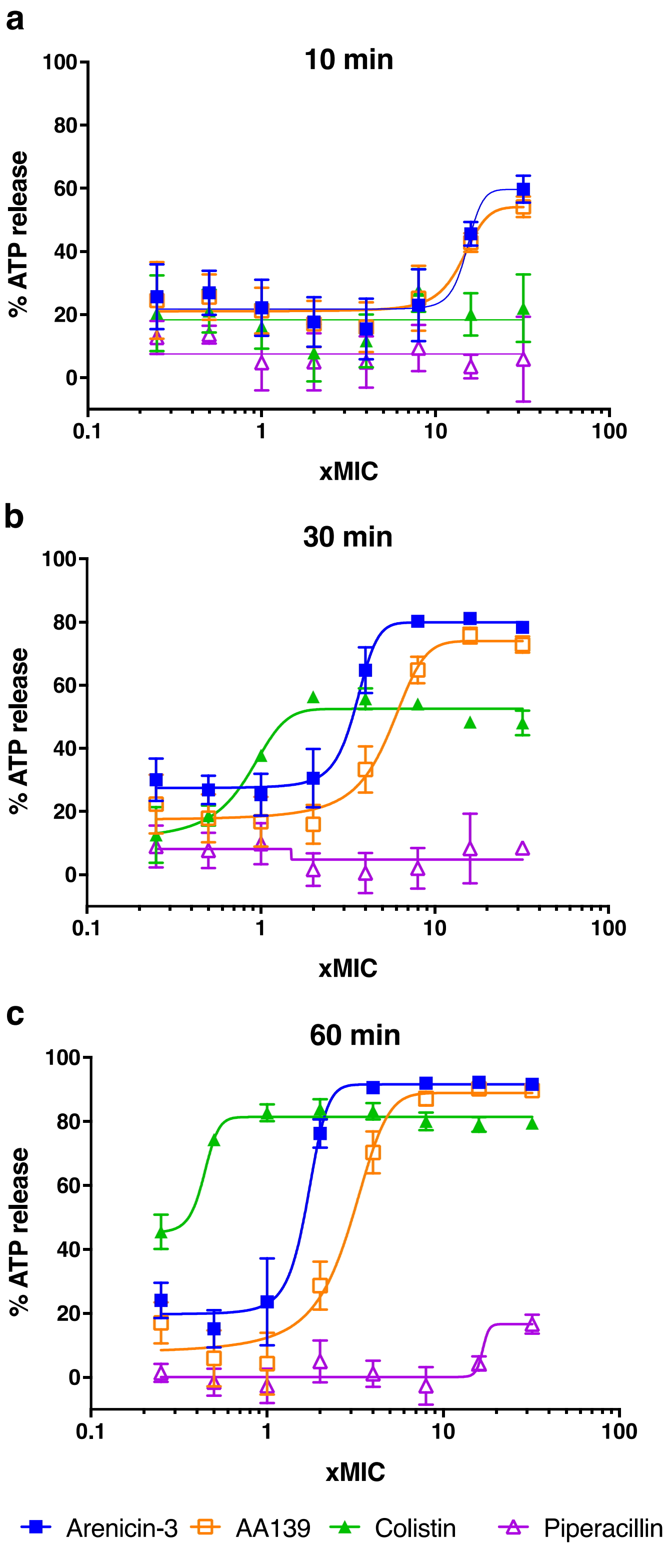

Supplementary Fig. 5: Extracellular ATP release. Arenicin-3 (closed blue squares), AA139 (open orange squares), colistin (closed green triangles), and piperacillin (open magenta triangles) induced release of ATP from *E. coli* K-12 MG1655. Exponential cells were incubated with drug and measured for extracellular ATP by luminescence after (**a**) 10 min, (**b**) 30 min, (**c**) 60 min incubation of cells with compound. Data analyzed using Prism 8 by nonlinear fit (log(inhibitor) vs. response, variable slope). Each data point is the mean of three independent experiments (n=3) with error bars displayed as SEM, y-axis is % ATP released (decreased) relative to untreated cells (0 × MIC) and x-axis is fold MIC applied. Source data are provided as a source data file.

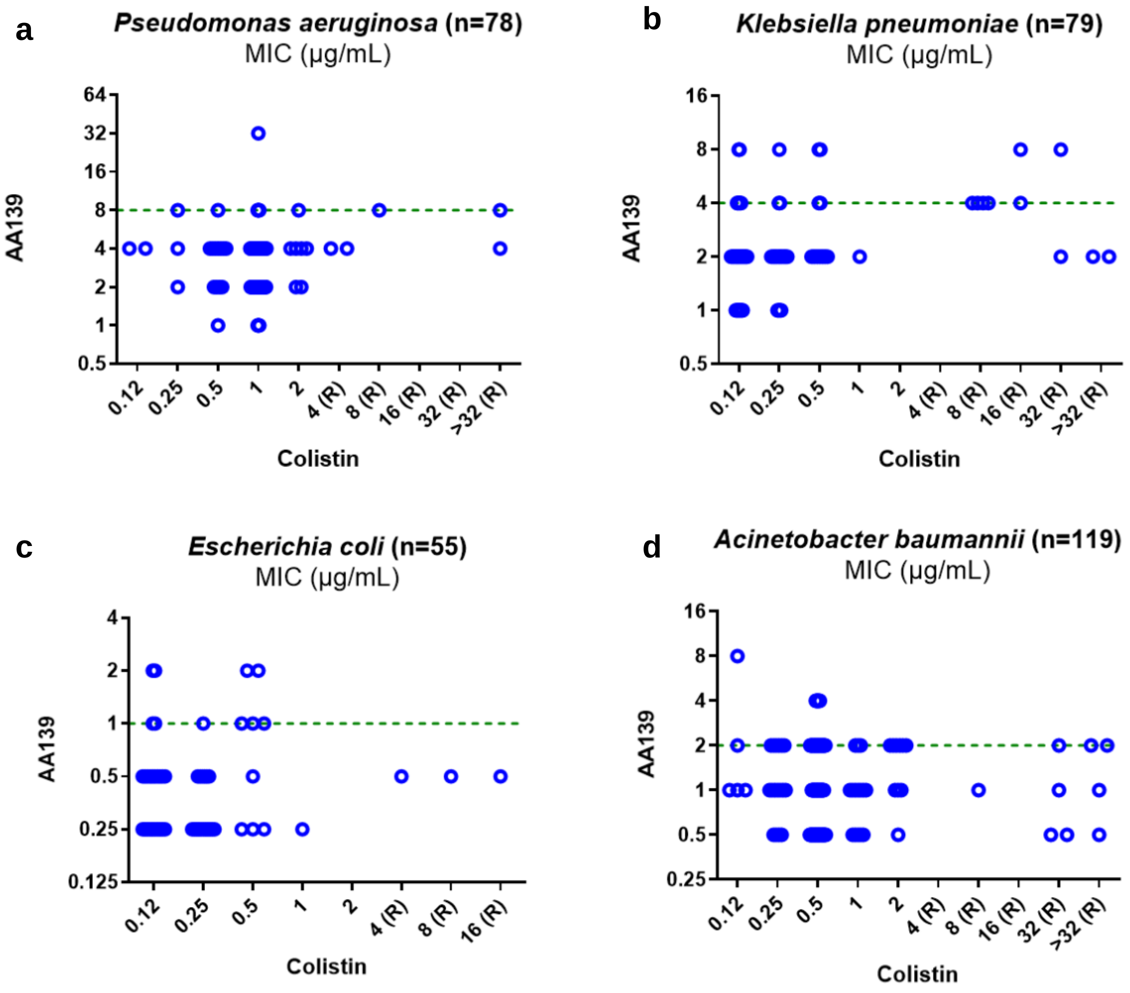

Supplementary Fig. 6: Assessment of cross resistance *in vitro*. Broth MIC values of AA139 and colistin for clinical US isolates of (**a**) *P. aeruginosa*, (**b**) *K. pneumoniae*, (**c**) *E. coli* and (**d**) *A. baumannii*. The green dotted horizontal line represent the MIC_{90} of AA139 for each species. The isolates are known resistance phenotypes, see Supplementary Table 3. Source data are provided as a source data file.

>S2:Query_46567:264273-264908 NODE_3_length_348865_cov_62.3445
TTATTTTTTCTCTTCCAGAGTGATTTTCTGTTGAGAAATCGATTTCAGTTGCGCAGTCAGGCCGTCGATACCTTT
GGTACGCAGCAGCGTTCCCCACTCGTTTTGTTTGGTGGTGATCATACTGACGCCTTCAGCAATCATGTCGTAAGC
CTGCCAGTTGCCCGTCTGGGAGTTTTTACGCCACTGGAAGTCCAGACGCACCGGCGGACGGCCATTCGGGTCAAT
AATGGTAACGCGAATCGGCACAATGGTTTTATCACCCAGCGGCTGTTCTGGCGCAATCTGATAGGTTTGACCGTG
ATACATCGCCAGCGCCTGACCGTAAGCCTGCTTCAGGTACTCACGGAAAGCGGCAAAGTAAGCATCACGTTGAGC
TGGGGTCGCACTCTTGTAATACTGGCCCAGCACCAGCGCACCGGCGTATTTCACCTGTACGTATGGCAACAGTTC
CTGATCAACAATGGTACGCAGATAATCCGGGTTAGCCTGAATTTGCGGTTGCTCATTCTTCAGGCGATCGAACGT
TTTCTGCGCCGCCTCGTCCATCAGCTTATACGGATTGGTCTGGTCTGCCGCGGTTGCCGCACTCAGCGGTGCAAT
CACCAGCAAAGCGACCATCATTAAACGTTTAAACAT

>S2_ReverseComplement:
ATGTTTAAACGTTTAATGATGGTCGCTTTGC**T**GGTGATTGCACCGCTGAGTGCGGCAACCGCGGCAGACCAGACC
AATCCGTATAAGCTGATGGACGAGGCGGCGCAGAAAACGTTCGATCGCCTGAAGAATGAGCAACCGCAAATTCAG
GCTAACCCGGATTATCTGCGTACCATTGTTGATCAGGAACTGTTGCCATACGTACAGGTGAAATACGCCGGTGCG
CTGGTGCTGGGCCAGTATTACAAGAGTGCGACCCCAGCTCAACGTGATGCTTACTTTGCCGCTTTCCGTGAGTAC
CTGAAGCAGGCTTACGGTCAGGCGCTGGCGATGTATCACGGTCAAACCTATCAGATTGCGCCAGAACAGCCGCTG
GGTGATAAAACCATTGTGCCGATTCGCGTTACCATTATTGACCCGAATGGCCGTCCGCCGGTGCGTCTGGACTTC
CAGTGGCGTAAAAACTCCCAGACGGGCAACTGGCAGGCTTACGACATGATTGCTGAAGGCGTCAGTATGATCACC
ACCAAACAAAACGAGTGGGGAACGCTGCTGCGTACCAAAGGTATCGACGGCCTGACTGCGCAACTGAAATCGATT
TCTCAACAGAAAATCACTCTGGAAGAGAAAAAATAA
>Translate:MFKRLMMVAL**L**VIAPLSAATAADQTNPYKLMDEAAQKTFDRLKNEQPQIQANPDYLRTIVDQEL
LPYVQVKYAGALVLGQYYKSATPAQRDAYFAAFREYLKQAYGQALAMYHGQTYQIAPEQPLGDKTIVPIRVTIID
PNGRPPVRLDFQWRKNSQTGNWQAYDMIAEGVSMITTKQNEWGTLLRTKGIDGLTAQLKSISQQKITLEEKK-

>S3:lcl:Query_6089:236851-237486 NODE_4_length_321443_cov_59.9315
ATGTTTAAACGTTTAATGATGGTCGCTTTGC**G**GGTGATTGCACCGCTGAGTGCGGCAACCGCGGCAGACCAGACC
AATCCGTATAAGCTGATGGACGAGGCGGCGCAGAAAACGTTCGATCGCCTGAAGAATGAGCAACCGCAAATTCAG
GCTAACCCGGATTATCTGCGTACCATTGTTGATCAGGAACTGTTGCCATACGTACAGGTGAAATACGCCGGTGCG
CTGGTGCTGGGCCAGTATTACAAGAGTGCGACCCCAGCTCAACGTGATGCTTACTTTGCCGCTTTCCGTGAGTAC
CTGAAGCAGGCTTACGGTCAGGCGCTGGCGATGTATCACGGTCAAACCTATCAGATTGCGCCAGAACAGCCGCTG
GGTGATAAAACCATTGTGCCGATTCGCGTTACCATTATTGACCCGAATGGCCGTCCGCCGGTGCGTCTGGACTTC
CAGTGGCGTAAAAACTCCCAGACGGGCAACTGGCAGGCTTACGACATGATTGCTGAAGGCGTCAGTATGATCACC
ACCAAACAAAACGAGTGGGGAACGCTGCTGCGTACCAAAGGTATCGACGGCCTGACTGCGCAACTGAAATCGATT
TCTCAACAGAAAATCACTCTGGAAGAGAAAAAATAA
>Translate:MFKRLMMVAL**R**VIAPLSAATAADQTNPYKLMDEAAQKTFDRLKNEQPQIQANPDYLRTIVDQEL
LPYVQVKYAGALVLGQYYKSATPAQRDAYFAAFREYLKQAYGQALAMYHGQTYQIAPEQPLGDKTIVPIRVTIID
PNGRPPVRLDFQWRKNSQTGNWQAYDMIAEGVSMITTKQNEWGTLLRTKGIDGLTAQLKSISQQKITLEEKK-

>S4:lcl:Query_5581:236742-237377 NODE_4_length_321334_cov_74.1834
ATGTTTAAACGTTTAATGATGGTCGCTTTGC**G**GGTGATTGCACCGCTGAGTGCGGCAACCGCGGCAGACCAGACC
AATCCGTATAAGCTGATGGACGAGGCGGCGCAGAAAACGTTCGATCGCCTGAAGAATGAGCAACCGCAAATTCAG
GCTAACCCGGATTATCTGCGTACCATTGTTGATCAGGAACTGTTGCCATACGTACAGGTGAAATACGCCGGTGCG
CTGGTGCTGGGCCAGTATTACAAGAGTGCGACCCCAGCTCAACGTGATGCTTACTTTGCCGCTTTCCGTGAGTAC
CTGAAGCAGGCTTACGGTCAGGCGCTGGCGATGTATCACGGTCAAACCTATCAGATTGCGCCAGAACAGCCGCTG
GGTGATAAAACCATTGTGCCGATTCGCGTTACCATTATTGACCCGAATGGCCGTCCGCCGGTGCGTCTGGACTTC
CAGTGGCGTAAAAACTCCCAGACGGGCAACTGGCAGGCTTACGACATGATTGCTGAAGGCGTCAGTATGATCACC
ACCAAACAAAACGAGTGGGGAACGCTGCTGCGTACCAAAGGTATCGACGGCCTGACTGCGCAACTGAAATCGATT
TCTCAACAGAAAATCACTCTGGAAGAGAAAAAATAA
>Translate:MFKRLMMVAL**R**VIAPLSAATAADQTNPYKLMDEAAQKTFDRLKNEQPQIQANPDYLRTIVDQEL
LPYVQVKYAGALVLGQYYKSATPAQRDAYFAAFREYLKQAYGQALAMYHGQTYQIAPEQPLGDKTIVPIRVTIID
PNGRPPVRLDFQWRKNSQTGNWQAYDMIAEGVSMITTKQNEWGTLLRTKGIDGLTAQLKSISQQKITLEEKK-

Summary: S3 and S4 have a mutation (nonsynonymous) at nucleotide 32 (T>G) compared to S2. Amino acid change at position 11 (L>R).

Supplementary Fig. 7: Genetic analysis of *E. coli* ATCC 25922 20 day serial/generational passaged isolate. MlaC gene compared for parent strain (S2), 20-day passaged isolate (S3) and 20-day +3 arenicin-3 free passages (S4). Sequences deposited as NCBI bioproject PRJNA511334 https://www.ncbi.nlm.nih.gov/bioproject/?term=PRJNA511334.

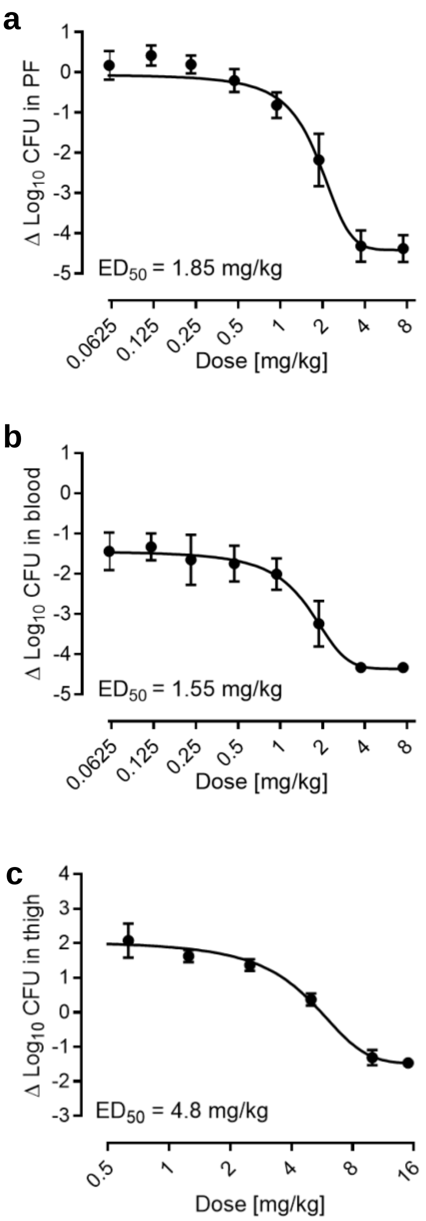

Supplementary Fig. 8: Dose response and ED_{50} of AA139 treatment following *E. coli* AID#172 MDR infection in murine models. (**a)** Bacterial load in peritoneal fluid (n=3 per dose group), 5 h post treatment, single i.v. dose 1h post infection in the peritoneum, (**b)** bacterial load in blood (n=3) from same experiment as peritoneal fluid, (**c)** bacterial load in the thigh (n=5) from separate experiment, compound dosed twice i.v. at 1 h and 6 h post intramuscular infection in the thigh, with CFU determined 24 h post first compound dose. PF: peritoneal fluid. Error bars are SEM. Source data are provided as a source data file.

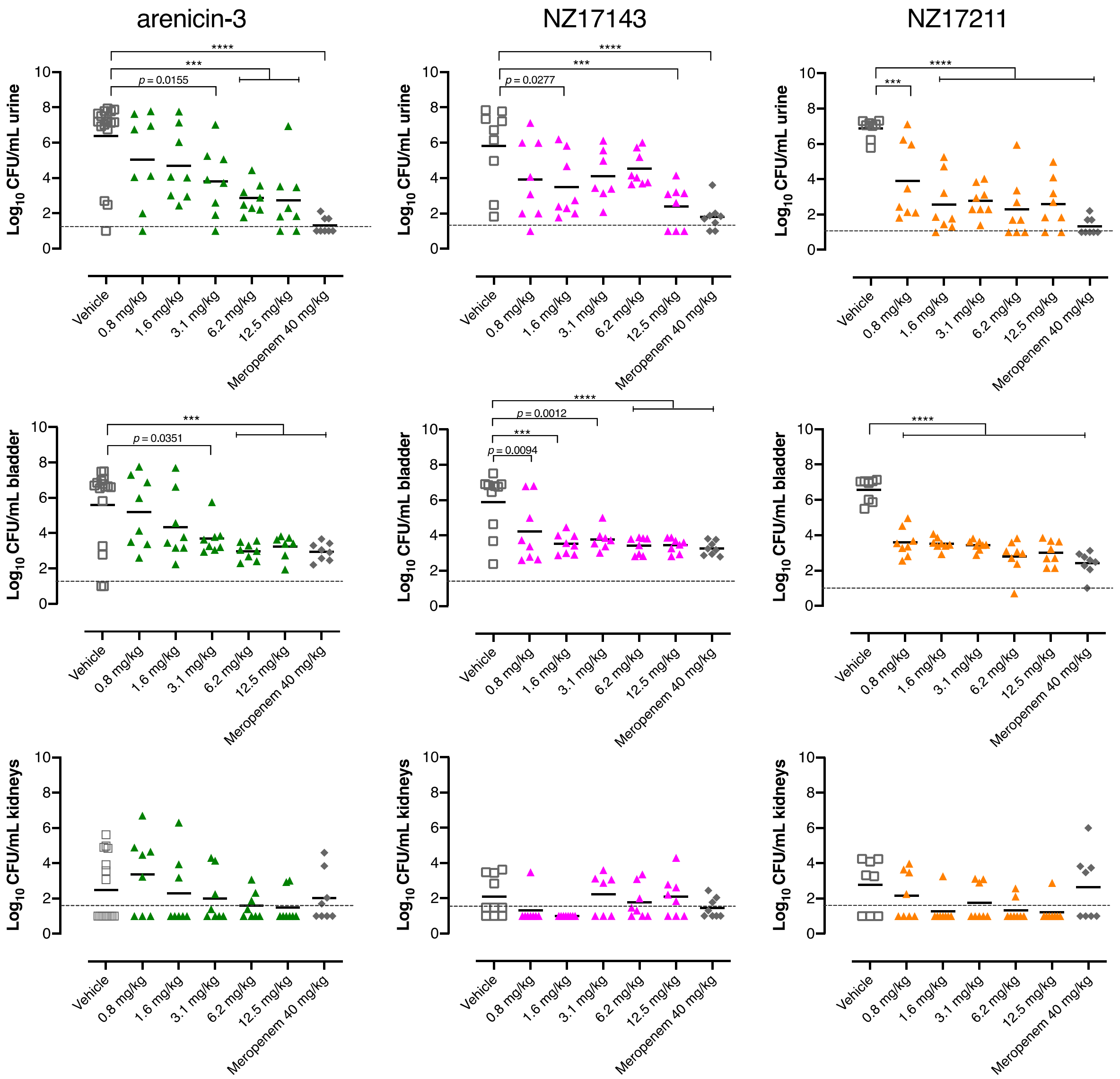

Supplementary Fig. 9: *In vivo E. coli* UTI (ESBL, DSA 443) murine model treated with arenicin-3 and its analogues. Arenicin-3 (left column, indicated as green triangles), NZ17143 (middle column, indicated as magenta triangles) and NZ17211 (right column, indicated as orange triangles) following twice daily i.v. dosing treatment of each peptide. CFU mL-1 bacterial burden read after 3 days in the urine, bladder and kidneys (from top to bottom for each peptide). Dotted lines represent the detection limit (i.e. countable burden of CFU mL-1 in the sample). Horizontal bar indicates geometric mean burden of each treatment (n = 8 per group). Significance between means is calculated by one-way ANOVA analysis (Dunnett's multiple comparisons test) using Prism 8, where all differences between means with $p \leq 0.05$ are indicated: ***$p \leq 0.0005$, ****$P \leq 0.0001$. Source data are provided as a source data file**.**

Supplementary Table 1: Chemical shift of arenicin-3 in phosphate buffer pH 3.3 at 298 K.

Chemical Shift (ppm)#															
Residue	N	NH	Cα	Hα	Cβ	Hβ	Cγ	Cδ	Hγ	Hδ	Hε	Hζ	Hη	Nε	Nδ2
Gly1			43.43	3.96		3.96									
Phe2	120.59	8.6	57.27	4.99	40.98	3.25,				7.36	7.24	7.21			
Cys3	119	8.19	55.81	5.57	48.27	2.50, 2.85									
Trp4	122.85	9.24		4.86	31.8	3.33,				6.96	9.98, 7.11	7.29, 6.82	6.98	128.69	
Tyr5		8.7	57.69	5.14		2.71, 2.84				6.88	6.76				
Val6	123.94	8.92	60.9	4.36	33.82	1.66	20.99		0.94						
Cys7	123.6	8.77	55.14	5.77	47.53	2.44, 3.00									
Val8	119.47	9.17	59.38	4.64	35.8	2.27	21.42		0.93, 1.01						
Tyr9		8.69	57.56	5.11		2.72, 2.84				6.9	6.78				
Arg10	124.48	9.09	54.85	4.63	32.29	1.65, 1.78	26.93	43.49	1.54, 1.65	3.21	7.22	124.81			
Asn11	125.63	9.64	54.31	4.4	37.55	2.80, 3.10				7.64, 6.95					112.77
Gly12	102.63	8.58	45.33	3.68, 4.25											
Val13	120.36	7.72	61.09	4.38	34.55	2.11	18.88		0.95						
Arg14	123.26	8.63	55.3	4.15	30.68	1.31, 1.69	27.82	43.57	0.92, 1.22	3.03	7.09	124.48			
Val15	129.72	9.16	61.49	4.24	34.32	1.91	20.77		0.81, 0.92						
Cys16	122.93	8.59	54.88	5.79	48.49	2.60, 2.98									
Tyr17	121.35	9.16		4.89		2.91, 3.00				6.97	6.65				
Arg18	123.26	8.71	55.3	4.2	31.76	1.36, 1.63	27.79	43.55	0.88, 1.13	2.95	6.97				
Arg19	128.63	8.67	54.08	4.29	32.6	0.55, 0.94	26.71	43.21	1.21, 1.34	3.12	7.14	124.94			
Cys20	116.84	8.09	55.07	5.2	48.78	2.82, 2.97									
Asn21	121.55	8.75	51.86	4.91	39.3	3.28, 2.97				6.66, 7.71					110.79

#Chemical shift are relative to DSS (0 ppm)

Supplementary Table 2: Hydrogen-deuterium exchange of arenicin-3 for prediction of hydrogen bonds.

Residues with slowly exchanging amides			Hydrogen bond prediction
1 h (weak)	3-4 h (medium)	>24 h (strong)	
C3, C7, N11, G12, C16, C20, N21	F2, Y5, R14, R18	W4, V6, V8, V13, V15, Y17, R19	W4-R19 (HN=O)<br>V6-Y17 (HN=O)<br>V8-V15 (HN=O)<br>V13-R10 (HN-O)

Supplementary Table 3: Clinical isolate panels used in this study to determine MIC_{90} values.

US isolates (2010-2012)		
**Organism**	**Phenotype**	**N**
P. aeruginosa N=78	Non-MDR	41
	MDR	25
	IPM-resistant	12
K. pneumoniae N=79	Non-MDR/Non-ESBL	24
	ESBL/MDR/carbapenem-resistant	34
	IPM-resistant	15
	Tigecycline-NS	5
	NDM-1	1
E. coli N=55	Non-MDR/Non-ESBL	24
	ESBL/MDR/carbapenem-resistant	21
	IPM-resistant	9
	NDM-1	1
A. baumannii N=119	Non-MDR	69
	MDR/carbapenem-resistant	31
	IPM-resistant	19
**Worldwide isolates (2011-2013)**		
**Organism**	**Phenotype**	**N**
P. aeruginosa N=111	Non-MDR	84
	MDR	16
	XDR	11
K. pneumoniae N=116	Non-MDR	80
	MDR	28
	XDR	8
E. coli N=110	Non-MDR	102
	MDR	7
	XDR	1
A. baumannii N=108	Non-MDR	19
	MDR	20
	XDR	69

MDR, Multi-drug resistant; XDR, Extensively Drug resistant; NDM-1, New Delhi metallo-b-lactamase-1; ESBL, Extended spectrum beta-lactamase; IPM, Imipenem resistant.

Non-MDR = resistant to 0-2 classes; MDR = resistant to 3-4 classes; XDR = resistant to > 4 classes.

Supplementary Table 4: Arenicin-3 and its analogues assessed by antimicrobial activity against *E. coli* ATCC 25922 in the presence of human serum (50%) and lung surfactant (SURVANTA® 5%).

Peptide	*E. coli* ATCC 25922				
	MHB	Serum (50%)	SURVANTA ® (5%)	Serum (50%)	SURVANTA ® (5%)
	MIC [μg mL-1]			Fold-decrease in activity	
Arenicin-3	1	16	8	16	8
AA139	0.125	2	0.125	16	2
NZ17125	0.125	8	8	64	64
NZ17126	0.125	2	0.5	16	4
NZ17143	0.25	1	0.5	8	2
NZ17160	0.125	2	0.5	16	4
NZ17211	0.5	4	2	16	4
NZ17224	0.06	1	0.25	16	4
NZ17228	0.125	2	0.5	16	4
NZ17230	0.25	2	0.5	8	2

Survanta® - Human lung surfactant; n=2 to 6, values are the mode if one is available or median where no mode is available, where n=2 differs the higher value is recorded; where no data is present for a strain in the presence of 50% Serum, the strain did not grow in the assay conditions. Source data are provided as a source data file.

Supplementary Table 5: Serum and lung surfactant (SURVANTA®) BMD MICs [µg mL$_{-1}$].

Peptide/ Antibiotic	*E. coli* ATCC 25922 FDA control strain		*A. baumannii* ATCC 19636 type strain		*K. pneumoniae* ATCC 13883 type strain		*K. pneumoniae* ATCC 700603 ESBL, OXA-2/SHV-18		*K. pneumoniae* BAA-2146 NDM-1		*P. aeruginosa* ATCC 27853 control strain		*P. aeruginosa* FADDI-070 MDR, PmxR	
	Serum (50%)	Survanta (5%)	Serum (50%)	Survanta (5%)	Serum (50%)	Survanta (5%)	Serum (50%)	Survanta (5%)	Serum (50%)	Survanta (5%)	Serum (50%)	Survanta (5%)	Serum (50%)	Survanta (5%)
Colistin	≤0.03	≤0.06	≤0.031	0.125	≤0.03	0.125	0.125	0.125		0.06	0.25	0.25	0.25	≥64
Ciprofloxacin	≤0.03	≤0.03		1	≤0.03	0.25	0.125	1		>64	0.25	1	0.06	0.5
Trimethoprim	4	1		32	0.5	1	32	4		>64	>64	>64	≥64	>64
Arenicin-3	16	8	8	32	2	8	16	4		≥64	16	32	≥32	>64
AA139	2	0.125	4	2	0.5	0.5	8	0.125		2	8	0.5	8	8
NZ17125	8	8		>64	2	32	16	1		64	32	32	32	>64
NZ17126	2	0.5		16	0.5	1	16	2		16	16	2	16	16
NZ17143	1	0.5	4	4	0.25	1	4	0.5		4	8	1	16	8
NZ17160	2	0.5		2	0.5	0.5	4	8		2	16	0.5	16	1
NZ17211	4	2	8	8	1	2	8	64		8	8	2	8	16
NZ17224	1	0.25		8	0.5	0.5	4	0.25		8	16	1	8	8
NZ17228	2	0.5		2	0.5	0.5	8	64		2	16	0.5	8	4
NZ17230	2	0.5	4	4	0.25	0.5	8	8		4	4	0.5	8	8

Survanta® - Human lung surfactant; n=2 to 6, values are the mode if one is available or median where no mode is available, where n=2 differs the higher value is recorded; where no data is present for a strain in the presence of 50% Serum, the strain did not grow in the assay conditions. Source data are provided as a source data file.

Supplementary Table 6: Spontaneous frequency of resistance in Gram-negative bacteria following treatment with AA139 or colistin at 4× and 8× MIC.

Organism	Strain/Resistance description	AA139			Colistin		
		MIC*	4× MIC	8× MIC	MIC*	4× MIC	8× MIC
P. aeruginosa	UNT138-1	16	1.6×10^{-9}	6.7×10^{-10}	2	9.7×10^{-10}	$<3.2 \times 10^{-10}$
P. aeruginosa	ATCC 27853	8	1.1×10^{-9}	6.7×10^{-10}	2	1.0×10^{-9}	$<2.4 \times 10^{-10}$
K. pneumoniae	SSI#3010	16	1.1×10^{-10}	1.1×10^{-10}	1	1.2×10^{-7}	1.9×10^{-8}
K. pneumoniae	ST258	16	1.8×10^{-10}	$<1.8 \times 10^{-10}$	1	4.6×10^{-8}	4.7×10^{-8}
E. coli	AID#172	8	2.4×10^{-10}	$<2.4 \times 10^{-10}$	1	>TC	2.4×10^{-8}
E. coli	ATCC 25922	8	$<1.5 \times 10^{-10}$	$<1.5 \times 10^{-10}$	1	3.9×10^{-8}	1.8×10^{-9}

(*) = Noble agar dilution MIC (μg mL^{-1}) used to calculate 4× and 8× compound concentration for resistance experiment. >TC = Colonies Too numerous to Count.

Supplementary Table 7: Resistance induction in two strains each of *E. coli*, *K. pneumoniae*, *P. aeruginosa*, and *A. baumannii* in presence of arenicin-3 (top), or AA139 (bottom) data plotted as line graph (see Fig. 4) during 20 repeat passages and post three drug free passages.

			Arenicin-3 MIC (µg/mL)																						Fold Increase in MIC	
			Passage Number																							
**Organism**	**Phenotype**	**Strain ID**	**MIC**	**1**	**2**	**3**	**4**	**5**	**6**	**7**	**8**	**9**	**10**	**11**	**12**	**13**	**14**	**15**	**16**	**17**	**18**	**19**	**20**	**Post**	**Day 20**	**Post**
E. coli	ATCC	ATCC 25922	0.5	0.25	2	4	4	4	4	4	4	8	8	8	8	8	8	8	8	4	8	8	8	0.5	16	0
	ESBL/MDR	1920923	0.25	0.25	0.5	0.5	0.5	0.5	0.5	0.5	1	1	1	1	1	1	1	1	1	1	2	2	2	1	8	4
K.pneumoniae	susceptible	2802144	1	0.5	2	2	8	8	8	16	16	16	16	16	16	16	16	16	32	32	32	32	64	32	64	32
	ESBL/MDR	1929544	1	1	2	2	4	4	4	8	8	16	16	16	32	32	32	32	32	64	64	128	128	64	128	64
P. aeruginosa	ATCC	ATCC 27853	0.5	0.5	1	1	1	1	2	4	8	4	4	4	4	4	8	8	8	8	8	8	8	2	16	4
	MDR	2808698	0.5	0.5	1	1	2	2	4	4	4	4	4	4	4	4	4	8	4	4	4	4	4	4	8	8
A. baumannii	susceptible	2802270	0.12	0.12	0.25	0.25	0.25	0.25	0.25	0.25	0.25	0.5	0.5	0.5	0.5	0.5	0.5	0.5	0.5	0.5	0.5	0.5	0.5	0.25	4	2
	MDR	2810965	0.25	0.12	0.5	0.5	0.5	0.5	1	1	1	1	1	1	1	1	1	1	1	1	2	2	2	2	8	8

			AA139 MIC (µg/mL)																						Fold Increase in MIC	
			Passage Number																							
**Organism**	**Phenotype**	**Strain ID**	**MIC**	**1**	**2**	**3**	**4**	**5**	**6**	**7**	**8**	**9**	**10**	**11**	**12**	**13**	**14**	**15**	**16**	**17**	**18**	**19**	**20**	**Post**	**Day 20**	**Post**
E. coli	ATCC	ATCC 25922	0.5	0.5	1	2	4	2	2	2	2	4	2	4	4	4	4	4	8	4	16	8	4	0.5	8	0
	ESBL/MDR	1920923	0.25	0.25	0.5	0.5	1	0.5	0.5	0.5	0.5	0.5	1	0.5	0.5	0.5	0.5	1	2	2	4	2	2	1	8	4
K.pneumoniae	susceptible	2802144	1	1	2	1	2	4	4	4	4	4	8	8	8	8	16	16	16	16	32	32	64	16	64	16
	ESBL/MDR	1929544	1	1	2	8	8	16	16	32	64	32	64	64	64	64	64	64	128	64	64	64	128	64	128	64
P. aeruginosa	ATCC	ATCC 27853	0.5	0.5	2	4	2	4	2	2	2	4	8	8	8	4	4	8	16	8	16	16	8	4	16	8
	MDR	2808698	0.5	0.5	2	2	4	4	4	8	16	8	8	8	8	8	8	16	32	32	16	32	16	8	32	16
A. baumannii	susceptible	2802270	0.13	0.13	0.5	1	1	1	0.5	1	1	1	1	1	1	1	1	1	1	1	2	2	2	1	16	8
	MDR	2810965	0.25	0.25	2	1	2	1	2	2	2	2	2	4	4	2	2	4	8	8	8	16	8	4	32	16

Supplementary Table 8: IV Pharmacokinetics of AA139.

Species	Dose	Route	Regimen	C_{max}	AUC_{inf}	$t_{½}$	V_d	Study
	(mg kg^{-1})			(ng mL^{-1})	(ng*h mL^{-1})	(h)	(L kg^{-1})	
**mouse**	10	i.v.	bolus	16,200	24,917	3.2	0.91	Fidelta, SR-056-14
**mouse**	10	i.v.	2-h infusion	5,105	13,950	2.9	NR	Covance 8286218
**monkey**	10	i.v.	2-h infusion	10,050	40,800	2.4	0.87	Covance 8315974
**minipig**	10	i.v.	2-h infusion	8,681	36,660	3.1	1.20	Covance 8286205

Mean data, males & females combined, N = 2 to 4 per group.

Supplementary Table 9: AA139 PK parameters following 2 hour daily inhalation administration to male and female mice.

		Dose (mg kg^{-1}/day)	T_{max} [a](h)	C_{max} (ng mL^{-1})	$AUC_{(0-t)}$ (ng*h mL^{-1})
**Plasma**	Day 1	5	0.3	122	262
		20	0.3	952	2,590
		40	0.3	2,465	4,540
	Day 7	5	0.3	64	85
		20	0	467	1,382
**Lung (ng/g)**	Day 1	5	0	3,935	8,585
		20	0	7,691	14,411
		40	0	16,162	7,002
	Day 7	5	0	4,228	11,802
		20	0.5	15,133	66,791
**ELF**	Day 1	5	0.5	14,750	27,250
		20	1.3	8,245	39,850
		40	0	36,300	63,300
	Day 7	5	0.3	21,100	58,650
		20	0	42,350	137,000

[a]Time relative to end of 2-hour inhalation administration.
