# Supplementary material for: An amphipathic peptide with antibiotic activity against multidrug-resistant Gram-negative bacteria
Source: Nat Commun. 2020 Jun 23;11:3184. doi: 10.1038/s41467-020-16950-x (PMC7311426; doi:10.1038/s41467-020-16950-x)
Supplement: Supplementary file 2 — Description of Additional Supplementary Files [file 41467_2020_16950_MOESM2_ESM.pdf]

Description of Additional Supplementary Files

File Name: Supplementary Data 1
Description: Physicochemical properties of arenicin-3 ala scan, des- and designer analogues.

File Name: Supplementary Data 2
Description: BMD MICs [μg mL-1] of all arenicin-3 peptides.

File Name: Supplementary Data 3
Description: Organisms used in this study.

File Name: Supplementary Data 4
Description: TraDIS identified genes.

File Name: Supplementary Data 5
Description: AA139 *in vivo* toxicity and safety pharmacology studies.
