# Supplementary material for: An amphipathic peptide with antibiotic activity against multidrug-resistant Gram-negative bacteria
Source: Nat Commun. 2020 Jun 23;11:3184. doi: 10.1038/s41467-020-16950-x (PMC7311426; doi:10.1038/s41467-020-16950-x)
Supplement: Supplementary file 8 — Reporting Summary [file 41467_2020_16950_MOESM8_ESM.pdf]

nature research

Corresponding author(s): Matthew Cooper

Last updated by author(s): 2020/05/21

Reporting Summary

Nature Research wishes to improve the reproducibility of the work that we publish. This form provides structure for consistency and transparency in reporting. For further information on Nature Research policies, see our Editorial Policies and the Editorial Policy Checklist.

Statistics

For all statistical analyses, confirm that the following items are present in the figure legend, table legend, main text, or Methods section.

n/a	Confirmed	
☐	☒	The exact sample size (*n*) for each experimental group/condition, given as a discrete number and unit of measurement
☐	☒	A statement on whether measurements were taken from distinct samples or whether the same sample was measured repeatedly
☐	☒	The statistical test(s) used AND whether they are one- or two-sided<br>*Only common tests should be described solely by name; describe more complex techniques in the Methods section.*
☐	☒	A description of all covariates tested
☐	☒	A description of any assumptions or corrections, such as tests of normality and adjustment for multiple comparisons
☐	☒	A full description of the statistical parameters including central tendency (e.g. means) or other basic estimates (e.g. regression coefficient) AND variation (e.g. standard deviation) or associated estimates of uncertainty (e.g. confidence intervals)
☐	☒	For null hypothesis testing, the test statistic (e.g. *F*, *t*, *r*) with confidence intervals, effect sizes, degrees of freedom and *P* value noted<br>*Give P values as exact values whenever suitable.*
☒	☐	For Bayesian analysis, information on the choice of priors and Markov chain Monte Carlo settings
☒	☐	For hierarchical and complex designs, identification of the appropriate level for tests and full reporting of outcomes
☒	☐	Estimates of effect sizes (e.g. Cohen's *d*, Pearson's *r*), indicating how they were calculated

Our web collection on statistics for biologists contains articles on many of the points above.

Software and code

Policy information about availability of computer code

Data collection	No data collection code software was used in this study.
Data analysis	CCPNMR software, Talos program, CYANA 3.0, MolProbity 4.2, ExPASy ProtParam tool, Prism 8 software, StatsDirect statistical software v 2.7, Phoenix WinNonlin version 6.2, Bio-TraDIS pipeline, SPAdes v3.10.1, RAST

For manuscripts utilizing custom algorithms or software that are central to the research but not yet described in published literature, software must be made available to editors and reviewers. We strongly encourage code deposition in a community repository (e.g. GitHub). See the Nature Research guidelines for submitting code & software for further information.

Data

Policy information about availability of data

All manuscripts must include a data availability statement. This statement should provide the following information, where applicable:

- Accession codes, unique identifiers, or web links for publicly available datasets
- A list of figures that have associated raw data
- A description of any restrictions on data availability

NMR solution structure is deposited as PDB 5V0Y and BMRB 30259. TraDIS data is deposited in the European Nucleotide Archive (ENA) under study PRJEB3226. Genome sequences of serial passage isolates are deposited to National Center for Biotechnology Information (NCBI) as Bioproject PRJNA511334. The source data underlying Figs 2, 5a-c and 6, Tables 1 and 2, Supplementary Figs 2, 3, 5, 6, 8 and 9, and Supplementary Tables 4 and 5 are provided as a Source Data file.

Field-specific reporting

Please select the one below that is the best fit for your research. If you are not sure, read the appropriate sections before making your selection.

- [x] Life sciences
- [ ] Behavioural & social sciences
- [ ] Ecological, evolutionary & environmental sciences

For a reference copy of the document with all sections, see nature.com/documents/nr-reporting-summary-flat.pdf

Life sciences study design

All studies must disclose on these points even when the disclosure is negative.

Sample size	Sample size calculations are not performed for preclinical studies and rather, group size is kept as small as possible to yield quality data and is dictated by industry standards, not statistical analysis. Rodent PK studies typically include 3/group (3/sex/group if both males and females are used), increased to 4 or 5 if there is reason to believe the drug may exhibit high variability. Toxicology studies of relative short duration are almost always 5/sex/group for rodents and 2 or 3/sex/group for large animal species. Chronic studies include larger numbers of animals to account for attrition over time. Preclinical pharmacology studies vary more and typically an assessment is made the by investigator who established the model as to how many animals are necessary to produce an interpretable result; this is not a true statistical sample size calculation but more of an informal assessment of variability in the model, animal viability over the time course of the study, etc.<br>All in vivo study protocols used in this work stated, "animal numbers are as low as possible to obtain significant difference between groups, and no alternative methods are available for the study". This was accepted by the Ethics committees named below.<br>For example: The S6 guidance from ICH on preclinical safety testing of biologics states:<br>3.4. Number/gender of animals<br>The number of animals used per dose has a direct bearing on the ability to detect toxicity. A small sample size may lead to failure to observe toxic events due to observed frequency alone regardless of severity. The limitations that are imposed by sample size, as often is the case for non-human primate studies, may be in part compensated by increasing the frequency and duration of monitoring. Both genders should generally be used, or justification given for specific omissions.
Data exclusions	Data was not excluded.
Replication	All in vitro studies were repeated at minimum of 2 technical replicates of each 2 biological replicates on independent days with independent prepared samples and reagents, with all attempts of replication successful. For in vivo studies technical replicates (i.e. multiple animals were used in the one assay. Replication for in vivo studies was not conducted where significant difference was seen between positive controls and negative controls in order to conserve animal usage. Additionally, the use of a variety of in vivo models was used to increase the significance of the findings as well as a variety of species across the varied models.
Randomization	Randomization was used in the selection of animals to be used in each of the in vivo studies from cohorts specifically bred for research purposes. Randomization is not relevant to the rest of our studies as no population, clinical data or field studies where randomization is required was carried out. All work was direct experimental design and all output used in each set of analysis.
Blinding	All studies were carried out in a blind fashion.

Reporting for specific materials, systems and methods

We require information from authors about some types of materials, experimental systems and methods used in many studies. Here, indicate whether each material, system or method listed is relevant to your study. If you are not sure if a list item applies to your research, read the appropriate section before selecting a response.

Materials & experimental systems

n/a	Involved in the study	
x		Antibodies
	x	Eukaryotic cell lines
x		Palaeontology and archaeology
	x	Animals and other organisms
	x	Human research participants
x		Clinical data
x		Dual use research of concern

Methods

n/a	Involved in the study	
x		ChIP-seq
x		Flow cytometry
x		MRI-based neuroimaging

Eukaryotic cell lines

Policy information about cell lines

Cell line source(s)	All eukaryotic cell lines used in this study were sourced from the American Type Culture Collection (ATCC), including HEK-293 human embryonic kidney cells (ATCC CRL-1573), HepG2 liver hepatocellular carcinoma cells (ATCC HB-8065) and HK-2 human kidney proximal tubule cells (ATCC CRL-2190).
Authentication	Cell lines were authenticated by ATCC standard STR profiling guidelines.
Mycoplasma contamination	Cell lines were tested for Mycoplasma contamination using the MycoAlert Mycoplasma Detection Kit (Cat #LT07-118).
Commonly misidentified lines (See ICLAC register)	No commonly misidentified cell lines were used in the study.

Animals and other organisms

Policy information about studies involving animals; ARRIVE guidelines recommended for reporting animal research

Laboratory animals	Peritonitis, pneumonia and MDR UTI infection models - NMRI (Harlan) female mice 6-11 weeks; ESBL UTI infection model - OF-1 female mice 6-11 weeks; PK/Tox - CD1 male and female mice 6-11 weeks, cynomolgus monkey male and female 4-7 years, minipig male and female 19-22 weeks. Mice were housed in polysulfone cages with an acclimatization period of 7 days prior to experiments with air temperature maintained at 22 degree Celsius with 55% humidity with light-dark cycles of 12 hours light (these conditions appear in the methods section of the main text).
Wild animals	No wild animals were used in this study.
Field-collected samples	No field collected samples were used in this study.
Ethics oversight	Peritonitis and UTI murine models were performed at the Department of Microbiology & Infection Control, Statens Serum Institute, Copenhagen, Denmark, under approval by the National Committee of Animal Ethics, Denmark, and adhered to the standards of EU Directive 2010/63/EU. The mouse pneumonia model was performed by Evotec Ltd, United Kingdom, under UK Home Office Licenses and with local ethical committee clearance. Pharmacokinetic and toxicokinetic studies were performed at Fidelta Ltd, Croatia, under review by the institutional ethics committee (CARE-Zg), and Covance Laboratories UK and Muenster Germany (monkey IV PK) in accordance with the requirements of the Animals (Scientific Procedures) Act 1986 and a maintained local ethical review.

Note that full information on the approval of the study protocol must also be provided in the manuscript.

Human research participants

Policy information about studies involving human research participants

Population characteristics	All sourced blood for haemolysis studies was donated by the Australian Red Cross Blood Service and no patient information is supplied to the researchers of this project as per the agreement with the Australian Red Cross and The University of Queensland Human Ethics Approval, therefore population characteristics are unknown.
Recruitment	All sourced blood for haemolysis studies was donated by the Australian Red Cross Blood Service and no recruitment information is supplied to the researchers of this project as per the agreement with the Australian Red Cross and The University of Queensland Human Ethics Approval, therefore recruitment information is unknown.
Ethics oversight	The use of human blood (sourced from the Australian Red Cross Blood Service) for haemolysis assays was approved by the University of Queensland Institutional Human Research Ethics Committee, Approval Number 2014000031.

Note that full information on the approval of the study protocol must also be provided in the manuscript.
